# Multifunctional protein 4.1R regulates the asymmetric segregation of Numb during terminal erythroid maturation

**DOI:** 10.1016/j.jbc.2021.101051

**Published:** 2021-08-06

**Authors:** Shu-Ching Huang, Long V. Vu, Faye H. Yu, Dan T. Nguyen, Edward J. Benz

**Affiliations:** 1 Department of Medical Oncology, Dana-Farber Cancer Institute, Boston, Massachusetts, USA; 2 Department of Medicine, Brigham and Women's Hospital, Boston, Massachusetts, USA; 3 Department of Medicine, Harvard Medical School, Boston, Massachusetts, USA; 4 Department of Pediatrics and Genetics, Harvard Medical School, Boston, Massachusetts, USA; 5 Leukemia Program, Dana-Farber/Harvard Cancer Center, Boston, Massachusetts, USA

**Keywords:** protein 4.1R, Numb, NuMA, LGN, spindle orientation, asymmetric cell division, erythroid differentiation, alternative splicing, 4.1R, protein 4.1R, 4.1R^−ex(20+21)^, 4.1R without exons 20 and 21, 4.1R^WT^, wild type 4.1R, Ab, antibody, ACD, asymmetric cell division, bp, base pair, CTD, C-terminal domain, d, day, DAPI, 4,6-diamidino-2-phenylindole, DMEM, Dulbecco's modified eagle medium, E/E, two erythroblasts with both positive for α-hemoglobin, ERM, ezrin/radixin/moesin, FERM, 4.1/ezrin/radixin/moesin, IB, immunoblotting, IF, immunofluorescence staining, IP, immunoprecipitate, MBD, membrane-binding domain, MELC, murine erythroleukemia cell, MIgG, mouse IgG, nt, nucleotides, P/E, one daughter positive for nestin or α-hemoglobin, P/P, two progenitors with both positive for Nestin, PI, propidium iodine, PRR, proline-rich region, PTB, phosphotyrosine binding domain, PVDF, polyvinylidene difluoride, RIgG, rabbit IgG, SCD, symmetric cell division

## Abstract

The asymmetric cell division of stem or progenitor cells generates daughter cells with distinct fates that balance proliferation and differentiation. Asymmetric segregation of Notch signaling regulatory protein Numb plays a crucial role in cell diversification. However, the molecular mechanism remains unclear. Here, we examined the unequal distribution of Numb in the daughter cells of murine erythroleukemia cells (MELCs) that undergo DMSO-induced erythroid differentiation. In contrast to the cytoplasmic localization of Numb during uninduced cell division, Numb is concentrated at the cell boundary in interphase, near the one-spindle pole in metaphase, and is unequally distributed to one daughter cell in anaphase in induced cells. The inheritance of Numb guides this daughter cell toward erythroid differentiation while the other cell remains a progenitor cell. Mitotic spindle orientation, critical for distribution of cell fate determinants, requires complex communication between the spindle microtubules and the cell cortex mediated by the NuMA-LGN-dynein/dynactin complex. Depletion of each individual member of the complex randomizes the position of Numb relative to the mitotic spindle. Gene replacement confirms that multifunctional erythrocyte protein 4.1R (4.1R) functions as a member of the NuMA-LGN-dynein/dynactin complex and is necessary for regulating spindle orientation, in which interaction between 4.1R and NuMA plays an important role. These results suggest that mispositioning of Numb is the result of spindle misorientation. Finally, disruption of the 4.1R-NuMA-LGN complex increases Notch signaling and decreases the erythroblast population. Together, our results identify a critical role for 4.1R in regulating the asymmetric segregation of Numb to mediate erythropoiesis.

Hematopoietic stem cells and progenitors can produce more stem or progenitor cells through self-renewal and are also responsible for the life-long replenishment of all mature blood cell types by way of differentiation. Stem cells can balance renewal with commitment through the regulation of symmetric and asymmetric cell division (1). During symmetric cell division, a stem cell divides into two stem cells (symmetric renewal) that allow the expansion of progenitors or into two committed cells (symmetric commitment) that permit the generation of mature cells. During asymmetric cell division, a mother cell generates two daughter cells, allowing one daughter cell to remain a stem cell and the other to differentiate into a mature blood cell. Thus, the hematopoietic system can maintain a pool of stem cells while giving rise to a population of differentiated blood cells.

The fundamental mechanisms critical for asymmetric cell division are not hardwired but responsive to cell-extrinsic or cell-intrinsic cues (2). The distinct extracellular environments that define stem cell identity are called stem cell niches (3). In ‘‘extrinsic’’ mechanisms, the two daughters of the dividing stem cell are positioned either inside or outside of the stem cell niche, leading to distinct fates (3, 4). In ‘‘intrinsic’’ mechanisms, the fate determinants are polarized in the dividing stem cell, resulting in unequal distribution of these determinants upon division and two daughter cells with different fates (5). In both situations, the mitotic spindle must be properly oriented by aligning the cell division plane with preestablished cell-extrinsic or cell-intrinsic asymmetries in order to ensure the asymmetric segregation of cell fate determinants into only one of the two daughter cells after stem cell division (2).

Numb is the first identified intrinsic molecular determinant of cell fate. The expression of Numb isoforms is differentially regulated and plays different roles in proliferation and differentiation (6). Unequal inheritance of Numb was first correlated with different daughter cell fate during the differentiation of the sensory organ precursor of *Drosophila melanogaster* (7). In mammals, asymmetric Numb distribution is critical to produce daughter cells that acquire distinct fates in the developing mouse retina (8) as well as to produce mouse cerebral cortical stem cells and neuroblasts (9). Hematopoietic stem cells undergo asymmetric cell division where Numb is asymmetrically distributed into one daughter cell and the inheritance of Numb is associated with a more differentiated fate (10).

Numb can antagonize another cell fate determinant, Notch, to diversify the fates of sister cells. Numb acts as an intracellular inhibitor of the Notch signaling pathway to specify sibling neuron cell fates in the *Drosophila* MP2 cell lineage (11). Asymmetric inheritance of Numb in terminal divisions of retinal progenitor cells creates unequal Notch signaling activity in sibling cells, leading them to acquire distinct fates (8). The asymmetric localization of Numb in hematopoietic stem cells results in inhibition of Notch signaling (10). Thus, reciprocal negative regulation between Notch and Numb may determine the balance of symmetric and asymmetric cell division (10, 12).

Spindle positioning is a carefully regulated process that is coordinated by several pathways. During mitosis, cortical actins line the plasma membrane all over the cortex, and retraction fibers, composed of actin filaments, maintain the cell–substrate adhesions (13). The extracellular matrix linked to the retraction fibers forms as the cell rounds up and can control spindle positioning *via* forces that are transmitted through the plasma membrane (13, 14). Inside the plasma membrane, spindle positioning is believed to be directed by interactions between the plus-end of astral microtubules that originate from the spindle poles and an evolutionarily conserved cortical machinery, the Gαi-LGN-NuMA ternary complex, which exerts a pulling force on them (15, 16, 17, 18). The Gαi-LGN-NuMA complex anchors the motor protein dynein at the cell cortex and polarizes cortical force generators. The movement of cortically attached dynein on the astral microtubules in the direction of the centrosome produces pulling forces for proper spindle positioning (19).

Multiple proteins have been shown to guide the component of the ternary complex to the cell cortex. Since the ternary complex and dynein act at the cell cortex in proximity of the actin-rich cytoskeleton, several recent reports have highlighted how F-actin and numerous actin-associated proteins directly or indirectly influence the localization or activity of the ternary complex components. The actin-binding protein 4.1R (20) is a member of the FERM (4.1/Ezrin/Radixin/Moesin) protein family. The FERM domain is a common protein module involved in localizing proteins to the plasma membrane where they function as membrane–cytoskeletal linkers and regulators of multiple signaling pathways (21). 4.1R interacts with microtubules and regulates the organization, dynamics, and attachment of microtubules to the cell cortex (22, 23, 24). The C-terminal domain of 4.1R interacts with NuMA (25) and is essential for mitotic spindle and aster microtubule dynamics and organization *in vitro* (26). The interactions of 4.1R with microtubules and NuMA make it an excellent candidate for examining its functional role in spindle orientation. Although 4.1R has been implicated in spindle orientation (27, 28), the specific mechanisms involved have not been characterized.

In this study, we examined the role of 4.1R in modulating the unequal distribution of Numb in the daughters of MELCs that undergo DMSO-induced erythroid differentiation. We show that 4.1R is critical for retraction fiber formation and astral microtubule stability. 4.1R associates with LGN-NuMA-p150^Glued^ in the same complex. Gene replacement confirms that the 4.1R-NuMA-LGN-dynein/dynactin complex is necessary for regulating spindle orientation, in which interaction between 4.1R and NuMA plays an important role. Finally, disruption of the 4.1R-NuMA-LGN complex increases Notch signaling and decreases the representation of the late erythroblast population during erythroid differentiation. Together, our results offer new insights into the biological function of 4.1R in regulating the asymmetric segregation of Numb and suggest a molecular mechanism by which 4.1R influences erythroid differentiation.

## Results

### A switch in Numb isoform expression occurs during erythroid differentiation

**Figure 1:**
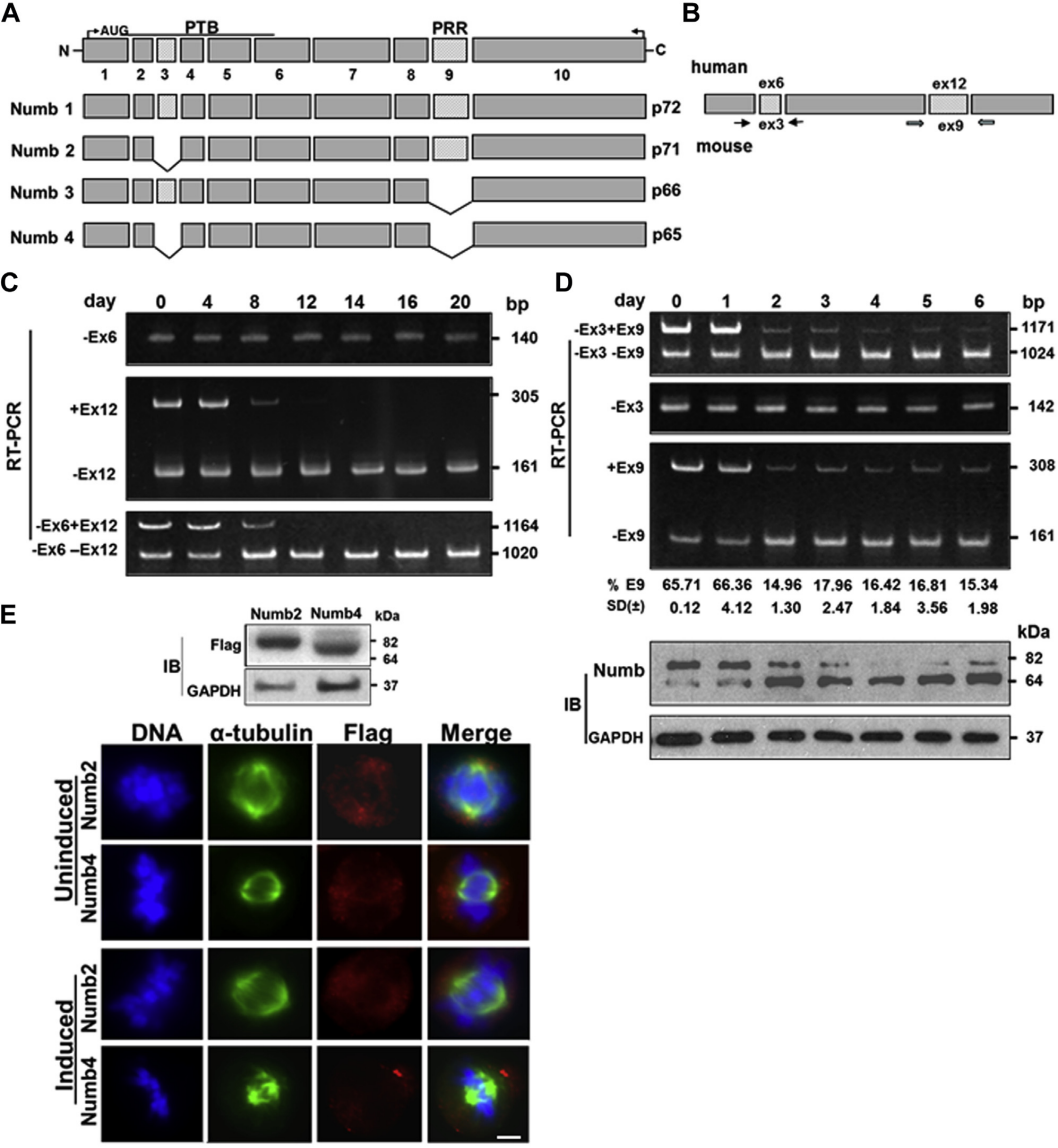
**A switch in Numb isoform expression occurs during erythroid differentiation.***A*, schematic diagram of mouse Numb and its domains. Constitutive exons are indicated as *dark gray boxes* and alternatively spliced cassettes are depicted as *light gray boxes*. Exon numbers are indicated. Alternative splicing generates four Numb isoforms containing different combinations of the presence or absence of exon 3 and exon 9. *B*, schematic representation of regions between exons 3 and 9 of mouse Numb or exons 6 and 12 of human Numb and the primer sets used in PCR. *C*, major Numb isoforms expressed in CD34^+^ cells undergo erythroid differentiation. RNAs isolated from day 0 to day 20 differentiating CD34^+^ cells were analyzed for exons 6 and 12 and combination of exons 6 and 12 expression by RT-PCR. Molecular markers base pair (bp) is provided at the right margin of the gels. *D*, major Numb isoforms expressed in differentiating MELCs. *Upper*, RNAs isolated from day 0 through day 6 DMSO-induced MELCs were analyzed for exon 3, exon 9, or a combination of exons 3 and 9 expression by RT-PCR. Molecular markers (bp) are provided. Exon 9 inclusion was calculated as the percent of total RNA products containing exon 9. All values are expressed as means ± SD from three independent experiments (n = 3) and presented at the *bottom* of each lane. *Lower*, lysates from the differentiating MELCs probed with an anti-Numb Ab. GAPDH served as a loading control. Molecular mass markers (kDa) are provided at the *right margin* of the blots. *E*, intracellular localization of Numb2 and Numb4 isoforms in uninduced and DMSO-induced MELCs. Numb isoforms with exon compositions as indicated in *A* were fused with Flag and transfected into MELC. *Upper*, Flag-Numb2 and -Numb4 expression in MELC probed with an anti-Flag Ab. *Lower*, Flag-Numb2 or -Numb4 in uninduced and induced MELCs were examined for their subcellular localization with an anti-Flag Ab as revealed by Zeiss microscopy. α-tubulin (*green*), Flag-Numb2 or -Numb4 (*red*), DAPI (*blue*). Bar, 5 μm. PRR, proline-rich domain; PTB, phosphotyrosine binding domain.

In mammals, the Numb gene produces four major transcripts that are generated from regulated alternative splicing of two exons; one is situated within the amino-terminal phosphotyrosine binding domain (PTB), and the other in the C-terminal proline-rich region (PRR) (Fig. 1*A*). In mouse Numb (NM_001136075.2), exon 3 with 33 nucleotides (nt) can be inserted in the PTB region and exon 9 with 144 nt, in the PRR region, distinguishing Numb1 (p72, +ex3/+ex9), Numb2 (p71, −ex3/+ex9), Numb3 (p66, +ex3/−ex9), and Numb4 (p65, −ex3/−ex9) isoforms. When aligned with mouse Numb, the human Numb (NM_001005743.1) gene has a similar overall coding structure and alternatively spliced exons but contains three additional exons located at the 5′-untranslated region. This aligns the mouse alternatively spliced exons 3 and 9 to human exons 6 and 12 (Fig. 1*B*).

The Numb protein is known to participate in asymmetric cell division (8, 9) and several signaling pathways (29) *via* alternative pre-mRNA splicing. Distinct Numb isoforms favor differentiation *versus* proliferation such as in the neuronal linages (30) and in the endocrine lineage of the developing pancreas (31). Furthermore, a switch in Numb isoforms is a critical step in cortical development (32). We first asked whether erythroid progenitors express specific Numb isoforms and whether isoform switches occur during the differentiation process.

We first analyzed Numb expression during induced erythroid differentiation of human CD34^+^ hematopoietic stem cells throughout a 20 day period (0, 4, 8, 12, 14, 16, and 20 days, corresponding to CD34^+^/BFU-E/CFU-E, proerythroblasts, basophilic erythroblasts, polychromatic erythroblasts, orthochromatic erythroblasts, reticulocytes, and erythrocytes) according to an established protocol (33). To characterize the exon composition of Numb isoforms, CD34^+^ RNA at each stage was interrogated *via* a limiting PCR cycle amplification protocol (34) for the presence of exon 6, exon 12, and the commonly expressed region between exons 6 and 12 (Fig. 1*B*). Exon 6 was completely excluded in all samples (Fig. 1*C*). Two nearly equal intensities of RNA species, corresponding to exon 12-inclusion or -exclusion, were detected in d0 and d4 cells. The intensity of the exon 12-containing band was then drastically reduced, with ∼20% exon 12-inclusion and ∼80% exon 12-exclusion in d8 cells. Exon 12-including mRNA was further reduced to 0% in d12 cells; this observation persisted throughout later stages of differentiation. Exon 12 is largely included in early differentiating cells and is mostly skipped in late erythroid cells. These results suggest that undifferentiated CD34^+^ cells express isoforms Numb2 and Numb4. In a manner similar to that seen in other tissues, there is a differentiation stage-specific shift of Numb forms (31, 32) from Numb2 to Numb4 isoforms during human erythroid differentiation.

The study of mitosis in primary CD34^+^ cells from humans is hampered due to the low mitotic index of CD34^+^ cells, making it difficult to analyze the number of cells necessary for meaningful statistical analysis. The MELC line provides a suitable alternative. MELC has been widely used for erythroid differentiation studies because it undergoes a workable rendition of erythroid differentiation when induced with DMSO (35). Despite incomplete synchronization after induction, the majority of the cells proceed synchronously through the same differentiation stages (36). To further understand the function of Numb in erythroid differentiation, we measured the relative expression levels of exon 3 and exon 9 in mouse Numb mRNA isolated from DMSO-induced day 0 through day 6 differentiating MELCs.

In a manner similar to that seen in CD34^+^ cells, two RNA species that exclude exon 3, but either include or exclude exon 9, were detected in uninduced MELCs (Fig. 1*D*). A differentiation-dependent switch from exon 9 inclusion to exon 9 exclusion was apparent in d2 through d6 cells. Quantitative analyses demonstrated that exon 9 comprises approximately 65% of the d0 and d1 RNA, then diminishes to ∼16% from d2 through d6 (Fig. 1*D*). Thus, the progressive exclusion of exon 9 in MELC or exon 12 in CD34^+^ is the major hallmark of Numb expression during erythroid differentiation.

To confirm these observations, we also analyzed Numb protein isoform expression in differentiating MELC by western blotting. We detected two isoforms with molecular weights of 71 kDa and 65 kDa in d0 and d1 differentiated cells. As cells proceeded through differentiation, the 71 kDa form decreased while the 65 kDa form increased (Fig. 1*D*). The difference between Numb 71 kDa and 65 kDa corresponds to inclusion or exclusion of exon 9. In keeping with the RNA results, exon 9-containing protein expression constituted almost 70% to 75% of the undifferentiated and early-differentiated cells and declined to ∼10% in later stage differentiated cells (Fig. 1*D*).

Numb proteins are asymmetrically distributed within multipotential neuronal (11) and erythroid (10, 37) progenitors to establish distinct progeny upon mitosis. We examined whether Numb2 and Numb4 had distinct intracellular localization by introducing these forms into MELC (Fig. 1*E*, upper panel). Both forms were localized in the cytoplasm of the uninduced MELCs, regardless of whether exon 9 was present or omitted (Fig. 1*E*, lower panel). However, only Numb4, lacking exon 9, was targeted to just one pole of the dividing differentiated cells. Numb2, containing exon 9, was never detected (Fig. 1*E*, lower panel). As in developing pancreas and cortical development (31, 32), exon 9 skipping appears to accompany erythroid differentiation and lead to asymmetric distribution of a selective isoform, Numb4.

### Asymmetric localization of Numb occurs in close proximity to 4.1R and the NuMA-LGN-p150^Glued^ complex in mitotic MELC during DMSO-induced erythroid differentiation

**Figure 2:**
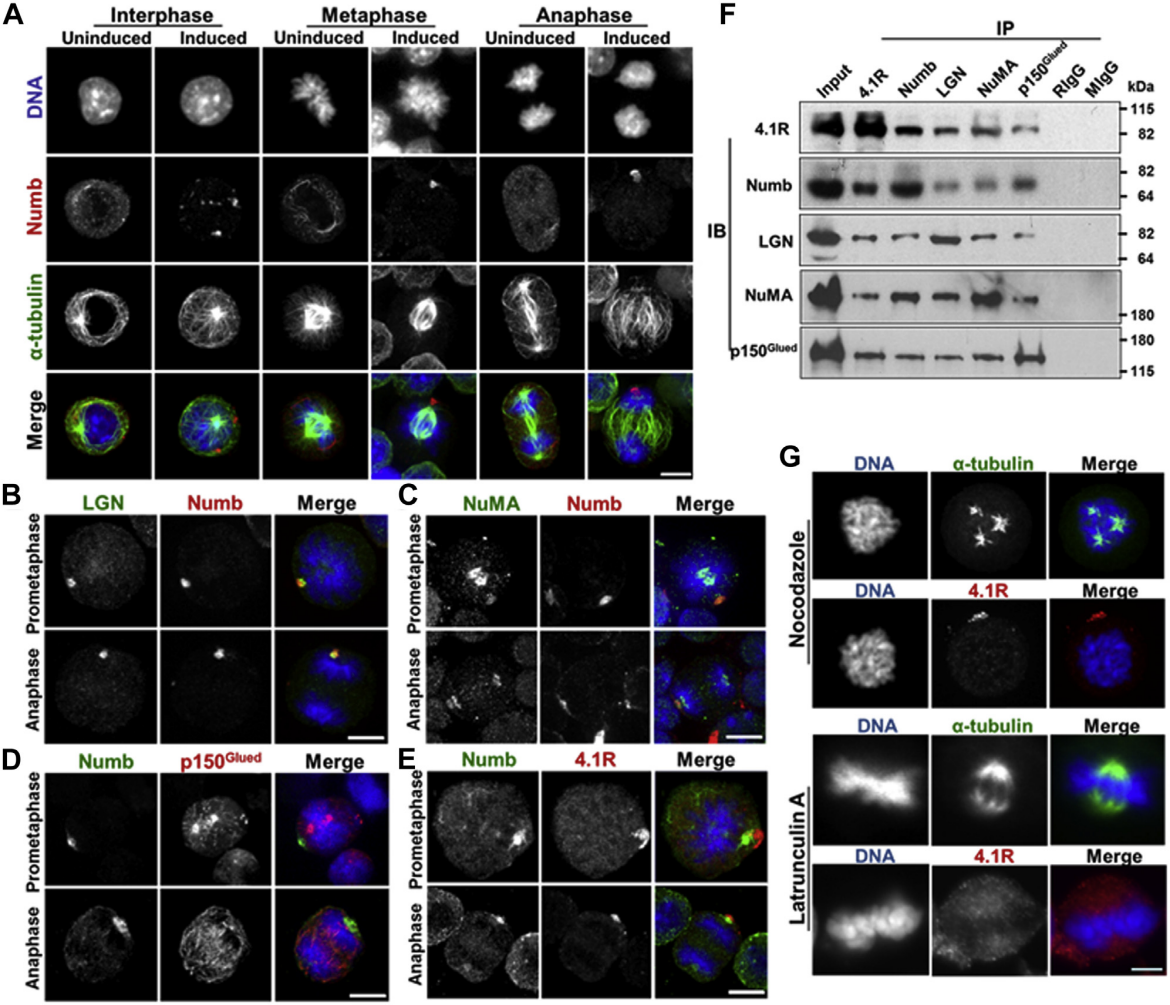
**Numb distributes asymmetrically near 4.1R and NuMA-LGN-p150**^**Glued**^**complex during mitosis in DMSO-induced MELCs.***A*, MELCs grown in normal medium (Uninduced) were switched to DMSO-containing medium (Induced). Cells at indicated phases of cell division were immunofluorescently stained for Numb (*red*) and α-tubulin (*green*) with its respective Ab and detected by Zeiss microscopy. Cells were counter stained with DAPI for DNA. Bar, 5 μm. *B*–*E*, induced MEL cells at prometaphase and anaphase were stained for LGN and Numb (*B*), NuMA and Numb (*C*), Numb and p150^Glued^ (*D*), or Numb and 4.1R (*E*) with each respective Ab, counterstained with DAPI, and detected by Zeiss microscopy. Bar, 5 μm. *F*, association of 4.1R, Numb, LGN, NuMA, and p150^Glued^ proteins in coimmunoprecipitation assays. Induced MELC lysates were precipitated (IP) with an anti-4.1R, anti-Numb, anti-LGN, anti-NuMA, anti-p150^Glued^, rabbit IgG (RIg), or mouse IgG (MIg) Ab. The input extracts and immunoprecipitates were examined by immunoblotting (IB) with each respective Ab. Molecular mass markers (kDa) are provided. *G*, induced MELCs were treated with nocodazole or latrunculin A and stained with anti-α-tubulin or anti-4.1R Ab and DAPI. Bar, 5 μm.

We compared the localization of Numb in uninduced and induced MELC during cell division. In interphase of the uninduced MELC, a fiber-like structure of Numb was detected in the cytoplasm (Fig. 2*A*). At metaphase and anaphase, Numb was detected in the cytoplasm and equally distributed to daughter cells (Fig. 2*A*). The localization of Numb in induced cells was different. At interphase, Numb was shown as a dot-like signal, close to the cell boundary. Numb localized to the cell cortex in the vicinity of one spindle pole at metaphase and unequally distributed to one daughter cell at anaphase (Fig. 2*A*). Both exogenous Numb4 and endogenous Numb can asymmetrically localize to one daughter cell in dividing MELC undergoing erythroid differentiation.

We asked how this asymmetric distribution of Numb is regulated. In *Drosophila* pl cells, Pins, the homolog of LGN in mammalian cells, localizes Numb by activating G protein signaling (38). Thus, we examined Numb localization relative to that of LGN in induced MELCs. Like Numb, LGN formed dot-like structure, which overlapped with Numb at the cell cortex of one dividing daughter cell from prometaphase through anaphase (Fig. 2*B*). Spindle orientation plays a key role in achieving an asymmetric outcome after stem cell division. A LGN-NuMA-dynein/dynactin complex is a conserved regulator of spindle orientation (17, 18, 27, 39, 40). Accordingly, we further examined the localization of NuMA and p150^Glued^ in differentiating MELCs. In addition to localizing to the spindle and spindle poles, NuMA also localized close to Numb at the cell cortex from prometaphase through anaphase (Fig. 2*C*). p150^Glued^ was shown to localize at kinetochores, microtubules, and spindle poles (41). p150^Glued^ localized at spindle poles appeared to attach to the surface of Numb at the cell cortex (Fig. 2*D*). We further examined the localization of 4.1R since 4.1R interacts with NuMA (25, 42). Like the cortical localization of LGN and NuMA, 4.1R abutted Numb at the cell cortex and asymmetrically distributed to one daughter cell in anaphase (Fig. 2*E*).

We subsequently examined whether 4.1R associated with the LGN-NuMA-dynein/dynactin complex and Numb in coimmunoprecipitation assays using induced MELC lysates and anti-4.1R, anti-Numb, anti-NuMA, anti-LGN, or anti-p150^Glued^ Abs. An 80 kDa 4.1R isoform expressed in MELCs. 4.1R antibody efficiently precipitated Numb, NuMA, LGN, and p150^Glued^ (Fig. 2*F*). The associations of Numb, LGN, NuMA, and p150^Glued^ were detected using each respective antibody. The two major HeLa 4.1R isoforms (135 kDa and 80 kDa) (Fig. S1*A*) also associated with the LGN-NuMA-dynein/dynactin complex (Fig. S1*B*). Furthermore, the NuMA-binding domain of 4.1R is critical for association with the complex (Fig. S1, *C*–*E*). These results suggest that 4.1R associates with Numb and the LGN-NuMA-dynein/dynactin proteins in the same complex through its NuMA-binding domain.

The cortical localization of LGN-NuMA-dynein/dynactin is maintained upon treatment with the microtubule dissociation agent nocodazole but is lost when treated with an F-actin depolymerizing drug latrunculin A (27). We examined whether the cortical localization of 4.1R is sensitive to either treatment. 4.1R localization to the cell cortex was not lost with nocodazole treatment but was lost after treatment with latrunculin A. These results suggest that 4.1R cortical localization is F-actin-dependent (Fig. 2*G*).

### 4.1R depletion induces mitotic arrest and its interaction with NuMA promotes the stability of astral microtubules and the association with the LGN-NuMA-p150^Glued^ complex

**Figure 3:**
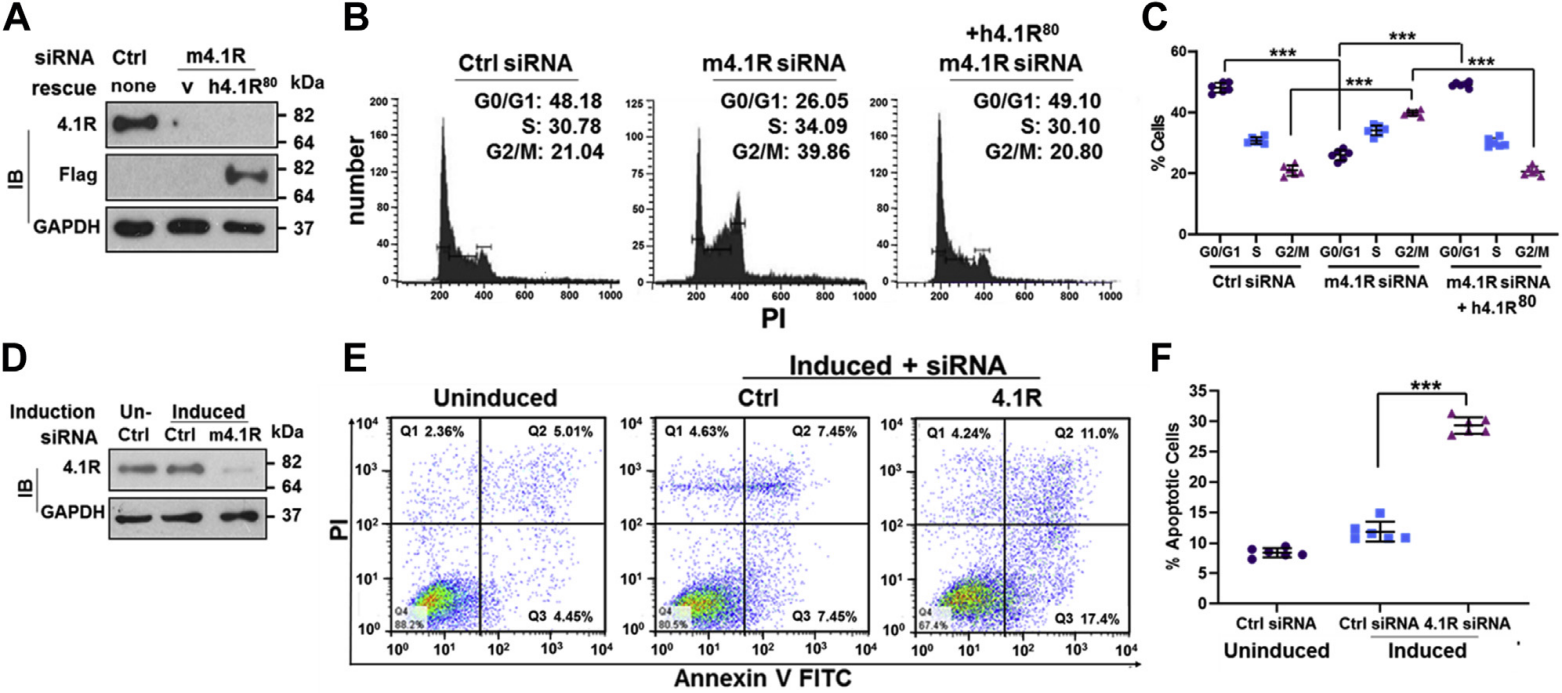
**4.1R depletion induced cell cycle arrest and increased apoptosis in DMSO-induced MELCs.***A*, efficiency of 4.1R depletion and rescue construct h4.1R^80^–Flag expression. Cell extracts of control siRNA, and m4.1R siRNA treated and transfected with or without h4.1R^80^-Flag were blotted with an anti-4.1R and anti-Flag Ab. GAPDH served as a loading control. Molecular mass markers (kDa) are provided. *B*, cell cycle distributions were assessed using propidium iodine (PI) staining with flow cytometry. Cell cycle distribution histograms of control siRNA or m4.1R siRNA treated and transfected without or with h4.1R^80^-Flag. *C*, quantification of G0/G1, S, and G2/M phases in control or m4.1R depleted with or without rescue construct h4.1R^80^-Flag. Six independent experiments were analyzed. *D*, efficiency of 4.1R depletion in uninduced or induced MELCs as indicated. *E*, representative apoptosis profiles assessed using annexin V-FITC and PI staining with flow cytometry. Cells from siRNA-treated samples, as indicated, were analyzed. *F*, quantification of apoptotic populations in control siRNA-treated uninduced and induced, or m4.1R-treated and -induced MELCs. Six independent experiments were analyzed. All values are expressed as means ± SD. Student *t* test for all graphs. ∗∗∗*p* < 0.0005.

The association of 4.1R with the LGN-NuMA-dynein/dynactin complex and its cortical localization (Fig. 2) imply a role for 4.1R in mitotic progression and spindle orientation. We analyzed the effect of 4.1R on cell cycle profiles of MELCs treated with a control or m4.1R siRNA with or without a rescue h4.1R construct. Treatment of 4.1R siRNA reduced 4.1R level to ∼5% while control siRNA did not affect 4.1R expression (Fig. 3*A*). The 4.1R rescue construct restored 4.1R expression in m4.1R siRNA-treated samples (Fig. 3*A*). All three MELC lines have similar S phase populations with ∼30% for the control, ∼34% for the 4.1R siRNA, and ∼30% for m4.1R siRNA depleted and h4.1R replenished cells. However, a significant increase in G2/M phase from ∼21% to ∼39% and decrease in G0/G1 phase from 48% to ∼26% were noted in the control and 4.1R depleted cells, respectively (Fig. 3, *B* and *C*). Rescue of 4.1R expression restored G0/G1 and G2/M phases to that of the normal cell cycle profile (Fig. 3, *B* and *C*).

To provide a more mechanistic insight into the mitotic regulation by 4.1R, we turned to the study of HeLa cells as they have been better characterized and more widely used to scrutinize several aspects of cell division, including spindle orientation (43). Time-lapse microscopy of HeLa stably expressing Histone H2B-GFP showed that 4.1R depletion delayed mitotic progression through prolongation of prometaphase or metaphase (Fig. S1*F*). 4.1R siRNA-treated cells increased duration of mitosis (Fig. S1*G*) and reduced normal chromosome morphology (Fig. S1*H*). Approximately 60% of 4.1R siRNA cells had aberrant chromosomes after prolonged arrest. Three types of aberrant chromosomes were noted: I, ∼38% eventually underwent apoptosis; II, ∼20% initiated anaphase in the presence of nonaligned chromosomes; III, 3% initiated anaphase but without successful chromosomes separation during cytokinesis (Fig. S1, *F* and *H*). These results show that cells depleted of 4.1R experienced delayed chromosome alignment and increased chromosome missegregation.

G2/M arrest is associated with apoptosis (44). We further analyzed whether an increase in the number of G2/M arrested cells also led to an increase in apoptosis in 4.1R depleted cells. DMSO induction of 4.1R depleted MELCs did not affect the effectiveness of 4.1R depletion as depletion reduced 4.1R level to ∼6% compared with the control (Fig. 3*D*). Flow cytometry analysis showed DMSO treatment slightly increased the apoptotic population from ∼8% in uninduced to ∼12% in induced cells. A significant increase in the apoptotic population to ∼29% occurred in 4.1R depleted MELCs (Fig. 3, *E* and *F*). These results suggest that chromosome instability due to 4.1R depletion resulted in cell cycle arrest and apoptosis in MELCs.

Proper spindle orientation is important for chromosome stability (45) and mitotic progression (46). Spindle positioning requires the actin cytoskeleton and retraction fibers to control mitotic centrosome positioning (13, 47). Mitotic actin-dependent pulling forces initiate from retraction fibers that link rounded mitotic cells to sites of cell matrix adhesion (13, 14, 48, 49). Interactions between the plus-end of astral microtubules and the cortical Gαi-LGN-NuMA ternary complex generate cortical spindle-pulling forces (15, 16, 17, 18).

When restriction fibers and astral microtubules were examined in mitotic HeLa cells, the control siRNA cells had “long” fibers while 4.1R siRNA cells had “short” fibers (Fig. S2*A*). The length of retraction fibers was partly restored in the presence of m4.1R^WT^ (Fig. S2*A*). 4.1R knockdown decreased the intensity of astral microtubule calculated as in Fig. S2*C* to ∼25% and the intensity was rescued up to 50% and 90% by expression of m4.1R^−ex(20+21)^ or m4.1R^WT^, respectively (Fig. S2, *B* and *D*). These results show that 4.1R is important for retraction fiber formation and astral microtubule stability (Fig. S2, *A* and *B*).

We have earlier shown that both the membrane-binding domain and C-terminal domain of 4.1R directly interact with microtubules (22) and that the C-terminal domain of 4.1R interacts with NuMA (42). Whether an overlapped interaction occurs among 4.1R, tubulin, and Numb requires further investigation. Nonetheless, these results suggest that 4.1R plays a role in astral microtubule stability and the interaction between 4.1R and NuMA has an additive effect in promoting the stability of astral microtubules.

### 4.1R is required for cortical localization of the LGN-NuMA-p150^Glued^ complex and for Numb’s position relative to the mitotic spindle in differentiating MELCs

**Figure 4:**
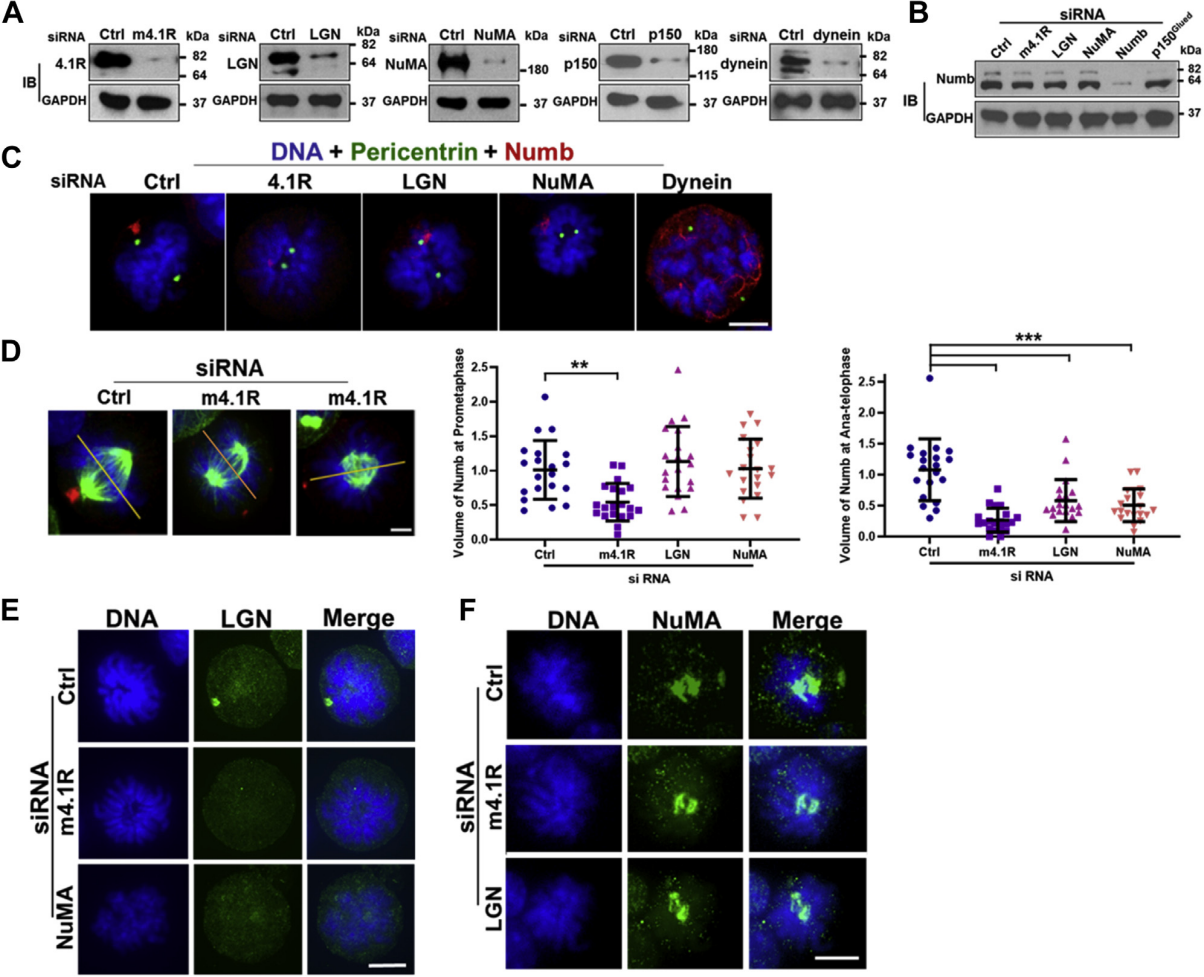
**4.1R, LGN, NuMA, and dynein/dynactin are required for Numb positioning relative to the mitotic spindle in induced MELCs.***A* and *B*, effectiveness of siRNA depletion analyzed by western blot. Cell lysates from siRNA-treated samples, as indicated, were interrogated with the Ab corresponding to each specific knockdown siRNA (*A*) or with Numb Ab (*B*). GAPDH served as a loading control. Molecular mass markers (kDa) are provided. *C*, effect of 4.1R, LGN, NuMA, or dynein depletion on cell cortex Numb shape and localization. Induced MELCs were stained with anti-Numb and anti-pericentrin Abs. DNA was stained with DAPI. Bar, 5 μm. *D*, the volume and localization of Numb relative to the equator (*yellow line*) in control and each protein knockdown line, as indicated, and stained with anti-Numb (*red*) and anti-α-tubulin (*green*) Abs. *Left panel*, example of control and 4.1R siRNA on the volume and localization of Numb. Bar, 5 μm. *Middle* and *Lower panels*, quantification of the volume of Numb in each indicated protein knockdown line during prometaphase (*Middle panel*) or ana-telophase (*Right panel*). n = 20 cells per group in each experiment. Three independent experiments were analyzed with similar results. All values are expressed as means ± SD. Student *t* test for all graphs. ∗∗∗*p* < 0.0005; ∗∗*p* < 0.005. Bar, 5 μm. *E* and *F*, depletion of each individual gene in 4.1R-NuMA-LGN complex prevents the localization of LGN (*E*) or NuMA (*F*) to the cell cortex. Induced MELCs stained with anti-LGN (*E*) or anti-NuMA (*F*) Ab as indicated and revealed with a Zeiss microscope. Bar, 5 μm.

To explore whether 4.1R, LGN, NuMA, and/or dynein/dynactin contribute to the cell cortical localization of Numb in differentiating MELCs (Fig. 2), we analyzed the effect of depleting expression of each protein on Numb expression and localization. The expression of 4.1R, LGN, NuMA, p150^Glued^, and dynein in MELCs was reduced to 4.5%, 8.6%, 6.2%, 5.2%, and 5.1% respectively (Fig. 4*A*). Numb expression was drastically and specifically reduced to ∼6.5% only by its siRNA (Fig. 4*B*).

Evaluation of the position of Numb relative to the mitotic spindle in each gene knockdown and DMSO-induced MELC line was next analyzed by immunofluorescent staining with anti-Numb and anti-pericentrin Abs. Both symmetric and asymmetric localization of Numb was observed in each treated mitotic cell except for dynein depleted cells. Dynein or p150^Glued^ (data not shown) depletion resulted in dispersed cytoplasmic localization of Numb (Fig. 4*C*). An intense round dot-like structure of Numb was localized adjacent to one spindle pole of the control cells. Asymmetrically distributed Numb signals were also detected in 4.1R, LGN, or NuMA depleted cells; however, Numb had an irregular shape with less intensity and localized at a wider angle to the spindle axis (Fig. 4*C*).

Examples of Numb localization in control and 4.1R knockdown cells are shown in Figure 4*D* (left panel). When the volume of Numb was quantified, a significant reduction was observed in 4.1R siRNA-treated cells but no differences were noted in LGN and NuMA cells in prometaphase (Fig. 4*D*, middle panel). The volume of Numb in 4.1R siRNA cells was ∼50% of control siRNA cells. As cells progressed into ana-telophase, all knockdowns exhibited significant reduction in Numb volume when compared with that of control siRNA cells (Fig. 4*D*, right panel). Numb in 4.1R siRNA cells was further reduced to ∼25%, while Numb in both LGN and NuMA siRNA cells was reduced to ∼50% compared with control (Fig. 4*D*, right panel). The effect of 4.1R on Numb distribution seems to occur at prometaphase and persist throughout the cycle, while LGN and NuMA exert their effects at a later cell cycle stage. Numb is dissociated from the cell cortex when treated with dynein or p150^Glued^ siRNA, suggesting that Numb is the cargo of a dynein/dynactin motor that transports it to the cell cortex. Together, these results suggest that 4.1R-LGN-NuMA-dynein/dynactin all influence the targeting of Numb to the cell cortex and the maintenance of the volume and morphology of Numb at various stages of mitosis.

When 4.1R and each component of the Gαi-LGN-NuMA-dynein/dynactin complex at the cell cortex were analyzed in HeLa cells, 4.1R and Gαi localized to the cell cortex of mitotic cells. Depletion of 4.1R did not alter cortical Gαi3 but depletion of Gαi3 completely abolished the cortical localization of 4.1R (Fig. S3*A*). 4.1R depletion also resulted in drastic reduction of the cortical localization of LGN (Fig. S3, *B* and *D*), NuMA (Fig. S3, *C* and *E*), and p150^Glued^ (Fig. S3, *C* and *F*). The decreased cortical localization of LGN, NuMA, and p150^glued^ was not rescued by m4.1R^−ex(20+21)^-mCherry that lacks the NuMA binding domain, but was restored in m4.1R^WT^–mCherry expressing cells (Fig. S3, *B* and *C*). These results suggest that 4.1R functions downstream of Gαi and upstream of LGN-NuMA-p150^Glued^ to allow for cortical localization of this complex and provide a functional role for 4.1R in recruiting LGN-NuMA-p150^Glued^ to the cell cortex through interaction between 4.1R and NuMA.

The relationships among 4.1R, LGN, and NuMA in their cortical localization were further investigated in differentiating MELCs. The effect of 4.1R or NuMA depletion on cortical localization of LGN was first examined. While an intense dot-like structure of LGN localized to the cell cortex of the control cells, there was no clear LGN cortical signal in 4.1R or NuMA depleted cells (Fig. 4*E*). Similarly, 4.1R or LGN depleted cells had reduced NuMA signal at the cortex (Fig. 4*F*). These results suggest that 4.1R is required for recruiting LGN and NuMA to the cell cortex and the presence of 4.1R-LGN-NuMA may be critical for cortical localization of Numb.

### Loss of 4.1R, LGN, or NuMA leads to spindle misorientation and reduces asymmetric cell division in induced MELCs

Mammalian cells lacking proper cortical localization of LGN-NuMA-dynein/dynactin exhibit defective spindle positioning (27, 43). We reasoned that spindle positioning might be compromised in cells depleted of 4.1R.

Studies of spindle orientation in control and 4.1R depleted HeLa cells showed that spindle poles reside in the same Z-plane in control cells but often reside in two different focal planes (Z1 or Z2) in 4.1R-deficient cells (Fig. S4*A*). Expression of m4.1R^−ex(20+21)^-mCherry did not restore the spindle poles to the same focal plane, but m4.1R^WT^-mCherry did (Fig. S4, *A* and *B*). Depletion of NuMA resulted in similarly expanded spindle angles (Fig. S4*B*, lower panel). Cells cultured on L-shaped fibronectin-coated micropatterns that forced cells to orient along the hypotenuse of the L-shape during mitosis (50) (Fig. S4*C*) also exhibited random spindle position in the subset of cells depleted of 4.1R (Fig. S4, *D* and *E*). Rescue with m4.1R^−ex(20+21)^-mCherry did not restore the α angle, but expression of m4.1R^WT^-mCherry recovered the α angle back to that of the controls (Fig. S4, *D* and *E*). Results from these studies imply that depleting 4.1R leads to severe spindle positioning defects.

**Figure 5:**
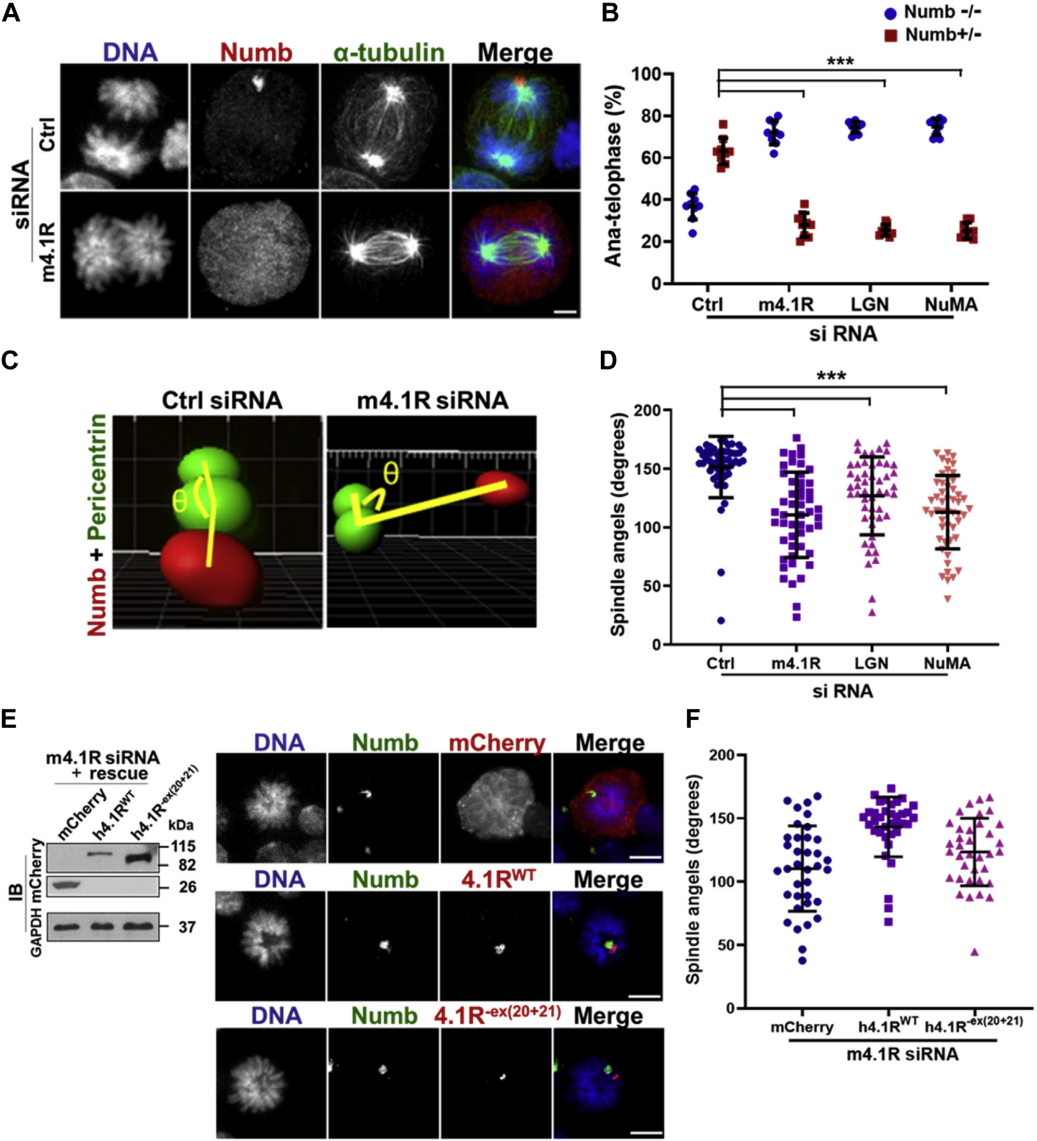
**4.1R, LGN, or NuMA depletion reduces asymmetric cell division in induced MELCs.***A*, symmetric and asymmetric segregation of Numb in control or siRNA-treated anaphase cells stained with anti-Numb (*red*) and anti-α-tubulin (*green*) Abs. Examples of control and 4.1R siRNA treated cells are shown. Bar, 5 μm. *B*, quantification of the effect of 4.1R, LGN, or NuMA depletion on symmetric Numb^−^/Numb^−^ and asymmetric Numb^+^/Numb^−^ cell division. n = 100 cells per group with three replicas in each experiment. Three independent experiments were analyzed. *C*, schematic diagram of 3D angle measurements for the position of Numb relative to mitotic spindle. 3D rendered images are accomplished from the induced MELCs stained with anti-Numb (*red*) and anti-pericentrin (*green*) Abs. The pericentrin signal that is closest to the Numb signal is set as the vertex of the angle. The centers of the Numb and pericentrin signals are selected for each angle measurement. Position of Numb relative to the mitotic spindle axis is obtained by measuring the angle (θ) shown in *yellow*. *D*, 4.1R, LGN, or NuMA depletion reduced the angles between Numb and spindle axis. Quantification of the angle measurements from the control and knockdown samples shown in *C*. n = 50 cells per group in each experiment. Three independent experiments were analyzed with similar results. *E*, 4.1R^WT^ rescues the angles between Numb and spindle axis. MELCs depleted of 4.1R were transfected with mCherry, h4.1R^WT^-mCherry, or h4.1R^−ex(20+21)^-mCherry. *Left panel*, expression of the exogenously expressed proteins was detected with an anti-mCherry Ab. *Right panel*, induced MELCs stained with anti-Numb (*green*) and anti-mCherry (*red*) Ab as indicated and revealed with a Zeiss microscope. Bars, 5 μm. *F*, quantification of the angle measurements from *E*. n = 35 cells per group in each experiment. Three independent experiments were analyzed with similar results. All values are expressed as means ± SD. Student *t* test for all graphs. ∗∗∗*p* < 0.0005.

None of protein depletions affected MELC entry into the cell cycle. Mitotic cells comprised approximately 25% to 30% of the population in induced control and in protein-specific knockdown cells. We went on to analyze whether the proportions of asymmetric and symmetric division were changed in mitotic cells depleted of each protein. Numb localizations in control and 4.1R knockdown cells are shown in Figure 5*A*. In metaphase and anaphase of control cells, ∼63.2% of the cells exhibited asymmetric Numb^+^/Numb^−^ localization. Only ∼36.7% of the control cells possessed symmetrically diffused Numb^−^/Numb^−^. In 4.1R knockdown cells, the asymmetric distribution was clearly reduced. Depletion of 4.1R, LGN, or NuMA all significantly decreased the percentage of Numb^+^/Numb^−^ asymmetric cell division in metaphase from ∼63.2% in the control to ∼26% in each gene knockdown. The same reduction in Numb^+^/Numb^−^ asymmetric distribution was also observed in anaphase and telophase (Fig. 5*B*). Thus, 4.1R, LGN, or NuMA depletion reduced asymmetric Numb cell division.

To evaluate the effect of gene depletion on the position of Numb relative to the spindle axis, we measured the angles as illustrated in Figure 5*C*. The centers of the Numb and pericentrin signals were selected for each angle measurement. The pericentrin signal closest to the Numb signal was set as the vertex of the angle. In control cells, the angles were highly regulated, with an average angle of ∼150° (Fig. 5*D*). Depletion of 4.1R, LGN, or NuMA randomized the angles, resulting in a spread of angles from 5 to 150° as illustrated schematically in Figure 5*C*. Quantification of the angles in each depleted cell revealed that more than 70% of the cells displayed an angle less than 150° (Fig. 5*D*). The mispositioning of Numb relative to the mitotic spindle could result from mistargeting Numb to the cell cortex or misorientation of the mitotic spindle. Since the distribution of cell fate determinants is not affected upon depletion of each ternary complex component (51), this suggests that misorientation of the mitotic spindle most likely results in altered Numb position relative to the spindle axis.

4.1R associates with Numb and the LGN-NuMA-dynein/dynactin proteins in the same complex (Fig. 2*F*) through the NuMA-binding domain of 4.1R exon 20 to 21 (Fig. S1*E*). We thus asked whether the effect of 4.1R depletion can be rescued by the expression of wild-type h4.1R^WT^-mCherry, or h4.1R^−ex(20+21)^-mCherry, which does not bind NuMA. Expression of h4.1R^WT^ restored the normal angle while h4.1R^−ex(20+21)^ expression only partially restored but did not completely rescue the angle to baseline (Fig. 5, *E* and *F*). These results imply that the interaction between 4.1R and NuMA is likely critical for proper spindle orientation and thus for proper Numb localization relative to the mitotic spindle.

### Asymmetric distribution of Numb is associated with asymmetric cell divisions and cell fate determination

Given the significant incidence of asymmetric Numb distribution in induced mitotic MELCs (Fig. 2), we investigated whether daughter cell fate is associated with the distribution of Numb. We established a “cell pair assay” to monitor MELC cell division at single-cell resolution to evaluate whether asymmetric Numb distribution is associated with asymmetric cell division during induction. Induced MELC were plated at low density, which gave rise to a single cell density in Cultureslides (BD) coated with ClonaCell FLEX (Stemcell Technologies). The majority of the first round of MELC divisions were completed by 22 h after cell attachment to the Cultureslide. Cells in each well were mapped 4 h after plating to record their location and again at 20 to 24 h to identify daughter cell pairs. The cells were fixed and immunofluorescently stained for Numb and DAPI. Both symmetric and asymmetric distributions of Numb were observed. In cases where both cells of a pair had detectable Numb, the intensity seemed to be similar for each cell. In other cases, Numb was detected strongly in only one daughter cell but not in the other. In yet another subgroup, both cells had no detectable amount of Numb. Such recordings were used to precisely identify Numb distribution in newly produced daughter cell pairs.

**Figure 6:**
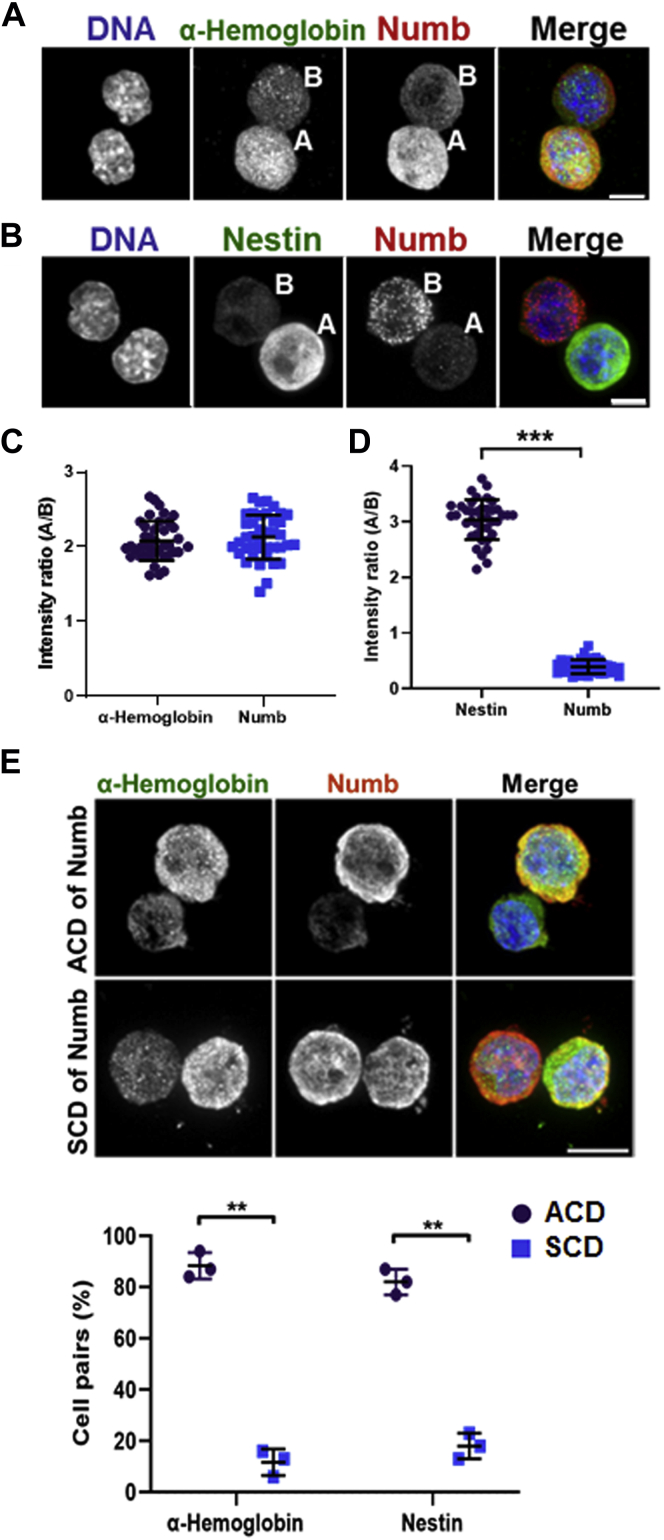
**Asymmetric Numb distribution is correlated with asymmetric cell division generating a progenitor cell and an erythroblast.** Induced MELCs were plated at low density, which gave rise to a single cell in Cultureslides (BD) coated with ClonaCell FLEX (Stemcell Technologies). Cells in each well were fixed and immunofluorescently stained as indicated 24 h after plating. *A*, MELC cell pair stained for Numb and α-hemoglobin. In the cell pair, the daughter cell that stained positive for α–hemoblobin is labeled with “A” and the other daughter cell with “B” for the ratio of intensity analyses in *C*. Bar, 5 μm. *B*, MELC cell pair stained for Numb and Nestin. In the cell pair, the daughter cell that stained positive for Nestin is labeled with “A” and the other daughter cell with “B” for the ratio of intensity analyses in *D*. Bar, 5 μm. *C*, intensity ratio of α-hemoglobin and Numb in cell pair shown in *A*. α-Hemoblobin or Numb intensities were obtained from fluorescence micrographs using ImageJ. The intensity ratio of cell A to cell B is calculated for α-hemoblobin or Numb. n = 40 cells per group in each experiment. Three independent experiments were analyzed with similar results. *D*, intensity ratio of Nestin and Numb in cell pair shown in *B*. The intensity ratio of cell A to cell B is calculated for Nestin or Numb. n = 40 cells per group in each experiment. Three independent experiments were analyzed with similar results. *E*, correlation between asymmetric distribution of Numb and cell fate determination in P/E pairs. *Upper panel*, example of daughter cell pair stained with anti-α-hemoglobin and anti-Numb Abs. Bar, 5 μm. *Lower panel*, quantification the association of Numb distribution and cell fate determination. Daughter cell pair stained with anti-α-hemoglobin and anti-Numb or anti-Nestin and anti-Numb Abs were quantified for asymmetric (ACD) and symmetric (SCD) Numb distribution in daughter pairs. n = 100 cells per group in each experiment. Three independent experiments were analyzed. All values are expressed as means ± SD. Student *t* test for all graphs. ∗∗∗*p* < 0.0005; ∗∗*p* < 0.005.

We analyzed the relationship between asymmetric Numb division and cell fates employing erythroid proliferation (favoring no differentiation) marker Nestin and the differentiation marker α-Hemoglobin. These markers can theoretically detect the following types of daughter cell pairs: two progenitors both positive for Nestin (P/P), only one daughter positive for Nestin or α-Hemoglobin (P/E), and two erythroblasts both positive for α-Hemoglobin (E/E). Cell pairs on Cultureslides were immunofluorescently stained 24 h after plating for the distribution of Numb and for their proliferation or differentiation status with an anti-Nestin or anti-α-Hemoglobin antibody. When α-Hemoglobin and Numb stained cell pairs were examined, the vast majority of the cells displayed codistribution of globin and Numb in one daughter cell and had minimally detectable signals in the other (Fig. 6*A*). In most of Nestin and Numb stained cell pairs, an inverse distribution of Nestin and Numb occurred as Nestin stained in one and Numb stained in the other daughter cell (Fig. 6*B*). These results suggest that Numb is associated with the differentiated daughter cell, while the absence of Numb represents the proliferating undifferentiated daughter.

Asymmetric division is defined as at least twofold difference in the intensity of Numb between paired daughter cells. The same criterion is also applied to α-Hemoglobin and Nestin for the quantification of the percentage of asymmetric division and cell fate determination. Among the majority of the P/E pairs, a twofold increase in intensity in the Numb and globin positive daughter cell was noted over the remaining daughter cell (Fig. 6*C*). Similarly, an approximately twofold increase in Nestin intensity was found in the daughter cell that stained with lower intensity for Numb (Fig. 6*D*).

Using this criterion, we quantified the association of Numb distribution and cell fate in differentiating MELCs. Since we do not have markers to distinguish whether the P/P or E/E cells are from either symmetric or asymmetric division, we only analyzed the P/E pairs. We analyzed cell pairs stained with Numb and α-Hemoglobin or Numb and Nestin (Fig. 6*E*, ACD, Asymmetric Cell Division; SCD, Symmetric Cell Division). Representative Numb and α-Hemoglobin staining of ACD and SCD cell pairs are shown in Figure 6*E* (upper panel). Among pairs positive for globin in one daughter cell, 88.4% were pairs with asymmetric Numb that codistributed with globin and 11.5% were pairs with symmetric Numb distribution in both daughters (Fig. 6*E*, lower panel). In pairs positive for Nestin in one daughter cell, 82.1% had asymmetric Numb distribution in the other daughter cell while 17.9% had symmetric Numb distribution in both cells (Fig. 6*E*, lower panel). A *t* test shows a highly significant association between asymmetric Numb distribution and asymmetric P/E fate. These results suggest that Numb asymmetric cell division is strongly associated with the production of two different daughter cell fates. The daughter cell inheriting Numb shifts toward differentiation while the other cell lacking Numb shifts toward proliferation. In conjunction with the results in Figure 5, *A* and *B*, depletion of each gene in the 4.1R-LGN-NuMA complex reduced the asymmetric distribution of Numb in daughter cell pairs, suggesting that the maintenance of spindle orientation by 4.1R-LGN-NuMA is critical for asymmetric Numb division and, thereby, cell fate decision.

### Depletion of Numb or 4.1R-LGN-NuMA proteins impedes MELC erythroid differentiation and increases Notch signaling

Depletion of each protein in the 4.1R-LGN-NuMA complex decreased the asymmetric division of the cell fate determinant Numb during erythroid differentiation (Fig. 5*B*). Numb distribution in daughter cells was strongly associated with differentiation (Fig. 6). We then further examined whether asymmetric cell division promotes erythroid differentiation. The MELC differentiation profile was monitored *via* flow cytometry in gene knockdown cells double-labeled for murine erythroid-specific TER119 and nonerythroid transferrin receptor (CD71) (36).

**Figure 7:**
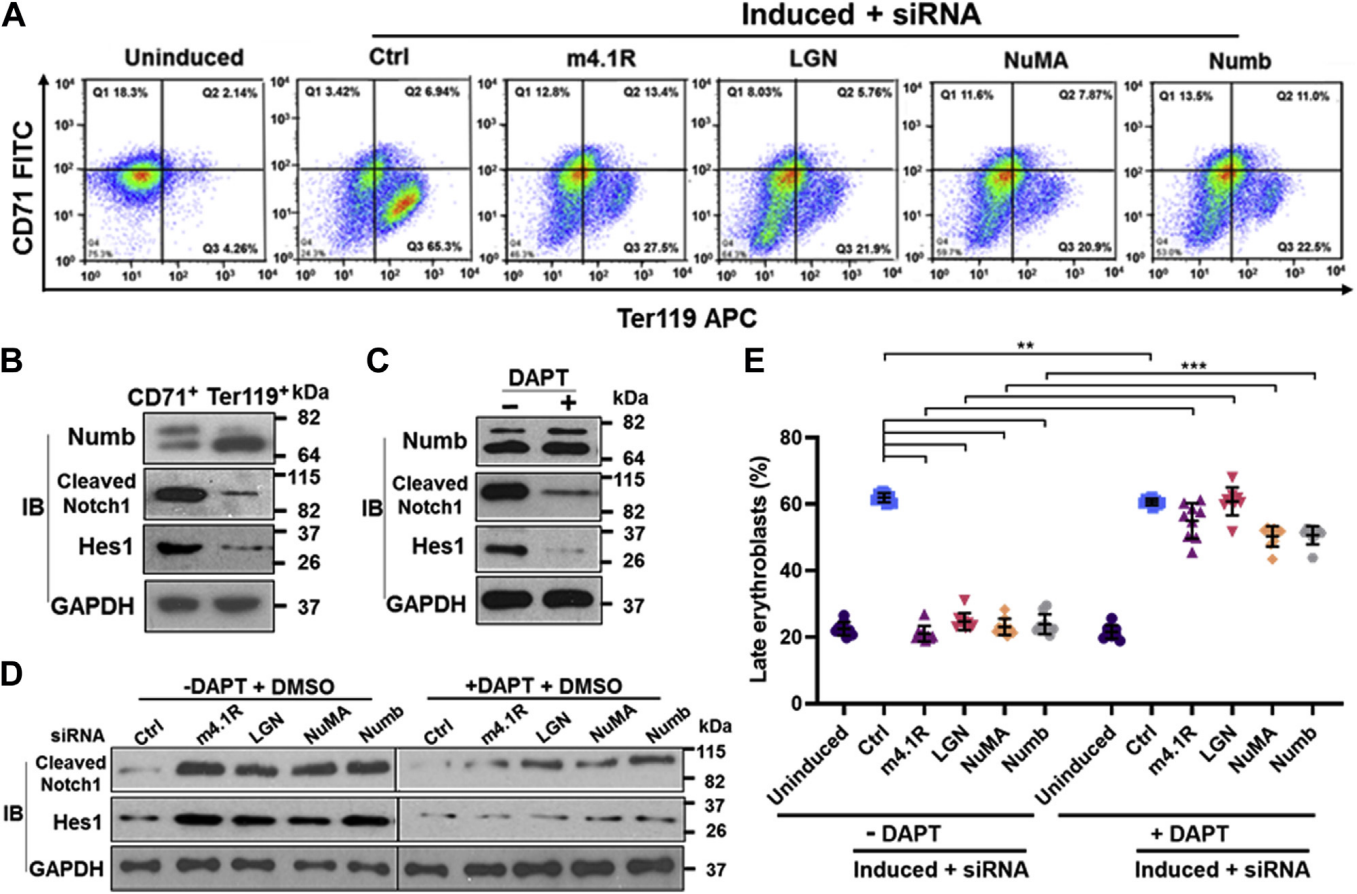
**Depletion of Numb or 4.1R-LGN-NuMA delays MELC maturation and increases Notch signaling.***A*, 4.1R, LGN, NuMA, or Numb depletion impaired erythroid differentiation. MELCs treated as indicated were double-stained for FITC-CD71 and APC-TER119 Abs and analyzed by flow cytometry. Representative flow cytometry profiles for each siRNA treatment as indicated. Regions defined by characteristic staining pattern: CD71^high^TER119^low^ (CD71^+^), CD71^high^TER119^high^ (CD71^+^/TER119^+^), and CD71^low^TER119^high^ (TER119^+^). The percentage of cells in each population is indicated. *B*, Numb, Notch1, and Hes1 expression analyzed in CD71^+^ and TER119^+^ cells sorted by fluorescence-activated cell sorter from induced MELCs. GAPDH served as a loading control. Molecular mass markers (kDa) are provided. *C*, DAPT treatment reduced Notch signaling. Western blot analysis of uninduced MELCs treated with or without DAPT for the presence of Numb, Notch1, and Hes1. GAPDH served as a loading control. Molecular mass markers (kDa) are provided. *D*, effect of DAPT on Notch signaling in induced MELCs treated with indicated siRNA. MELC treated with indicated siRNA in the presence or absence of DAPT was analyzed for the expression of Notch1 and Hes1. GAPDH served as a loading control. Molecular mass markers (kDa) are provided. *E*, quantification of erythroblasts in indicated gene knockdown cells treated or untreated with DAPT. (n = 9 flow cytometry profiles per group) All values are expressed as means ± SD. Student *t* test for all graphs. ∗∗∗*p* < 0.0005; ∗∗*p* < 0.005.

The undifferentiated cells contained mainly the CD71^low^Ter119^low^ and CD71^high^Ter119^low^ populations (Fig. 7*A*). MELC control siRNA treated and induced cells shifted from a less mature CD71^low^Ter119^low^ and CD71^high^Ter119^low^ to a more mature CD71^low^Ter119^high^ population that accounted for ∼65.3% of the total (Fig. 7*A*). The production of a mature population (CD71^low^Ter119^high^) was much less efficient and reduced to ∼27.5% for 4.1R siRNA cells (Fig. 7*A*). Depletion of LGN or NuMA had a similar effect and resulted in a ∼22% mature population in each knockdown (Fig. 7*A*). Moreover, depletion of Numb also reduced the mature population to ∼22.5%. Conversely, there was significant accumulation of the immature population (CD71^low^Ter119^low^) from 24% in the control to 46, 64, 60, and 53% in 4.1R, LGN, NuMA, and Numb knockdown cells, respectively. These results suggest a correlation between a higher degree of asymmetric distribution of Numb and better advancement in erythroid differentiation.

Notch and Numb play a role in balancing symmetric/asymmetric cell divisions (10, 12, 37). The subpopulations expressing activated Notch and Numb have different cell fates; Notch is critical in maintaining a proliferating progenitor population while Numb is important for differentiation (52). We analyzed the protein expression for cleaved Notch1, its downstream target Hes1, and Numb in less and more differentiated MELC using flow cytometry sorted CD71^high^Ter119^low^ (CD71^+^) and CD71^low^Ter119^high^ (Ter119^+^) cells (Fig. 7*B*). Similar to the results from unsorted cells in MELC differentiation (Fig. 1*D*), a differentiation switch in Numb isoform expression occurred as Numb2 decreased and Numb4 increased in the Ter119^+^ population (Fig. 7*B*). An actual increase in total Numb4 expression level was also observed in the differentiated Ter119^+^ population (Fig. 7*B*). Furthermore, an approximately fourfold decrease in Notch1 and Hes1 expression in Ter119^+^ cells occurred when compared with that of the CD71^+^ population (Fig. 7*B*). These results suggest that an inverse relationship between Numb isoform expression and Notch signaling is regulated in a temporal and spatial fashion during expansion and terminal erythroid differentiation phases of MELC induction.

The Notch pathway can be blocked with the γ-secretase inhibitor DAPT, which decreases levels of cleaved Notch1 (53). We examined the effect of Notch inhibition on cleaved Notch1, Hes1, and Numb expression in uninduced MELC. DAPT significantly affected the Notch signaling pathway as the cleaved Notch1 and Hes1 were reduced by 80%, but it had no effect on Numb levels (Fig. 7*C*).

The effect of Notch signaling on erythroid differentiation in induced MELCs treated with 4.1R, LGN, NuMA, or Numb siRNAs was then examined (Fig. 7, *D* and *E*). In the absence of DAPT, depletion of 4.1R-LGN-NuMA as well as Numb drastically increased the levels of cleaved Notch1 and Hes1 compared with that of control cells (Fig. 7*D*, −DAPT). These results suggest that Numb depletion can decrease its inhibitory effect on Notch signaling and results in increased Notch1 and Hes1 (Fig. 7*D*). Depletion of 4.1R, LGN, or NuMA reduced the volume and intensity of Numb (Fig. 4*D*) and increased symmetrical distribution of Numb (Fig. 5*B*), which may reduce the allocation of Numb to each daughter cell and result in increased Notch signaling. The increased Notch signaling pathway also resulted in increased proliferation and reduced erythroblast production in flow cytometric analyses (Fig. 7*E*, −DAPT). In the presence of DAPT, at least a threefold reduction in cleaved Notch1 and Hes1 was observed in each gene knockdown sample (Fig. 7*D*, +DAPT) and increased erythroblasts (Fig. 7*E*, +DAPT). These results show that erythroid expansion and terminal differentiation are at least partially regulated by an inverse relationship between Notch and Numb.

**Figure 8:**
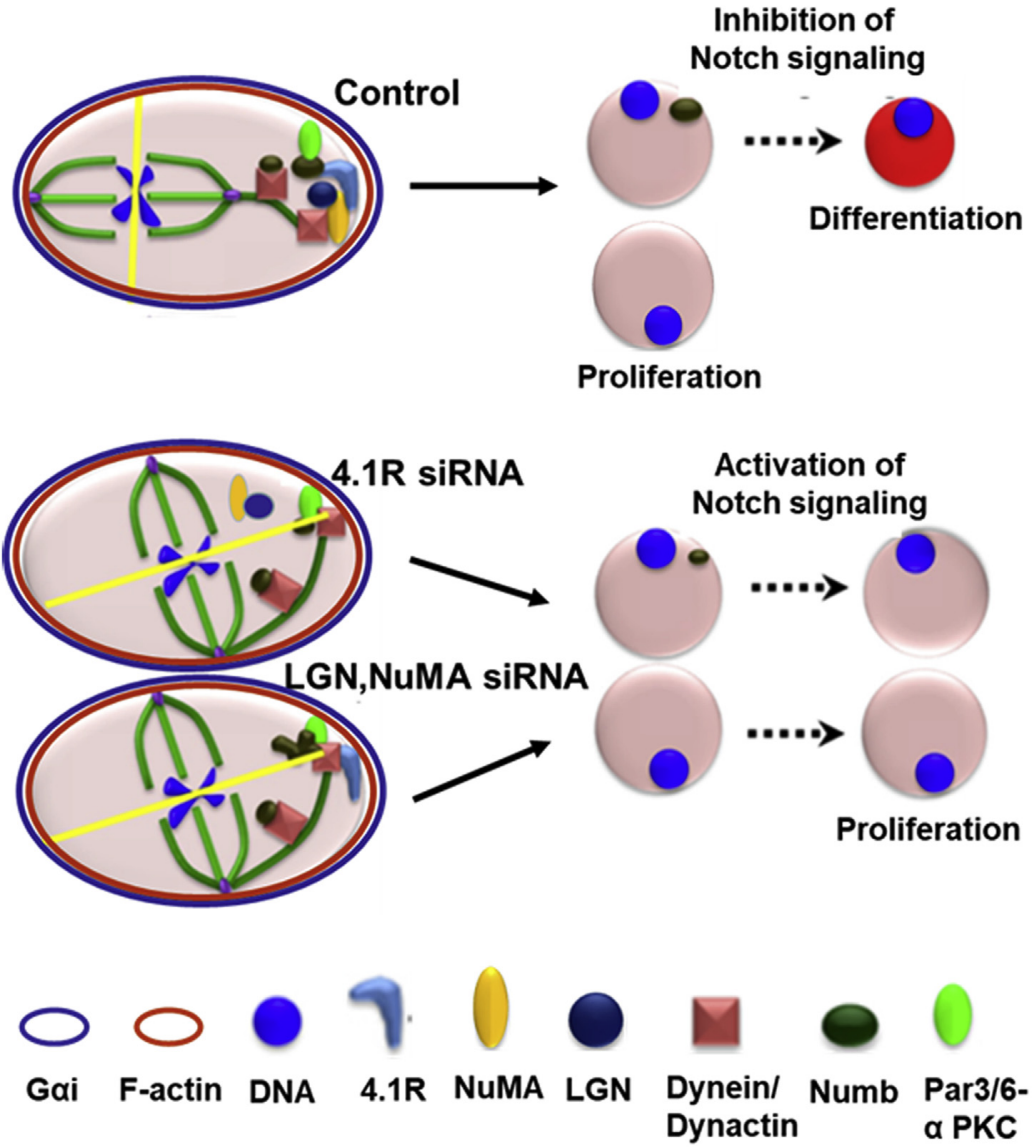
**Molecular model for 4.1R function in spindle orientation.** See text in Results for explanation.

Taken together, our data suggest a possible mechanism for 4.1R-mediated spindle orientation and erythroid differentiation (Fig. 8).

A Par3/6-atypical PKC complex establishes polarity (54). Cortical actin-binding by protein 4.1R reads the cell polarity and serves as an anchor to load LGN-NuMA-dynein/dynactin to the cell cortex through its direct interaction with NuMA. 4.1R also interacts and stabilizes astral microtubules. Thus, the 4.1R-NuMA-LGN-dynein/dynactin complex provides a cortical attachment site for astral microtubule to ensure the appropriate orientation of the mitotic spindle. Numb likely is the cargo of dynein/dynactin and is transported to the cell cortex, as cells treated with dynein or p150^Glued^ siRNA dissociate Numb from the cell cortex. The mitotic spindle is orientated toward the cell polarity so that Numb and other functional proteins can be asymmetrically distributed to one daughter cell after cell division. The daughter cell that inherits Numb moves toward differentiation while the other daughter cell that lacks Numb shifts to cell proliferation.

When 4.1R is depleted, LGN and NuMA are disconnected from the cell cortex, resulting in spindle misorientation. Dynein/dynactin can still be loaded to the cell cortex through its direct interaction with Par3. Hence, Numb can still be transported to the cell cortex by dynein/dynactin. However, Par3 alone is not sufficient to target Numb to the cell cortex without 4.1R-LGN-NuMA. When either LGN or NuMA is depleted, the spindle is misoriented, and 4.1R is still localized at the cell cortex. Although a normal amount of Numb is loaded to the cell cortex, Numb structure is abolished in the absence of LGN or NuMA. It appears that 4.1R, NuMA, and LGN are all necessary for the proper configuration and localization of Numb to the spindle pole of the differentiating daughter cell. The underlying biochemical interactions are under investigation.

Since spindle orientation sets up the division plane, misorientation of the mitotic spindle in 4.1R, LGN, or NuMA depleted cells could result in an incorrect division plane setup during early anaphase. This potentially results in certain Numb components not being able to be loaded into the cell cortex. Thus, these depletions significantly increase the ratio of symmetric distribution of Numb. Furthermore, knockdown of these genes reduces the size of Numb in the daughter cells and increases Notch signaling, which directly promotes cell proliferation and delays cell maturation.

## Discussion

One fundamental mechanism by which hematopoietic cells balance renewal of stem cells and differentiate into mature blood cells is *via* control of asymmetric cell division. The Gαi-LGN-NuMA protein complex links astral microtubules to the cell cortex and establishes the proper spindle orientation that is needed for asymmetric cell division. However, the molecular connections among these structures remain incompletely defined. Finding the components that link spindle microtubules to the cell cortex will improve our understanding of the regulation of spindle orientation. In this study, we show that the actin-binding protein 4.1R stabilizes astral microtubules and recruits LGN-NuMA to the cell cortex, thereby promoting interactions between the mitotic spindle and the cell periphery for spindle orientation. Our data provide a molecular framework for the complex interplay, mediated by 4.1R, in spindle orientation during asymmetric cell division. This in turn segregates the cell fate determinant Numb into one daughter cell and regulates erythroid differentiation.

The role of the FERM family proteins in cell division is credited to their ability to link actin filaments to the plasma membrane (20), stabilize microtubules at the cell cortex (55), and regulate LGN-NuMA cortical localization and spindle orientation (56). The ERM protein moesin binds to and stabilizes microtubules at the cell cortex through two conserved lysine residues (55). However, these lysine residues are conserved among ERM and ERM-related merlin but not found in other FERM family proteins. We have shown earlier that 4.1R interacts with microtubules through the FERM and CTD domains and plays a role in microtubule aster assembly (22). From the rescue experiments with 4.1R^WT^ and 4.1R^−ex(20+21)^ in this study, we further confirmed that both domains are required for astral microtubule stability (Fig. S1*E*). 4.1R also indirectly binds to microtubules through its binding to microtubule plus-end-tracking protein CLASP2 and regulates the attachment of microtubules to the cell cortex (24). Thus, 4.1R can directly or indirectly interact with microtubules and regulate microtubule dynamics. The stabilization of astral microtubules by 4.1R ensures microtubule extension to the cell cortex (Fig. S2) and hence interaction with cortical sites, a prerequisite for orientation of the spindle.

Even though FERM family proteins have been shown to be involved in the cortical localization of the LGN-NuMA force generator complex and spindle orientation (55, 56), the specific functional role of 4.1R in these processes is not well characterized. In this study, we showed that 4.1R functions downstream of Gαi and upstream of LGN-NuMA-dynein/dynactin (Fig. S3, *A*–*C*), and its depletion markedly impaired both the cortical localization of LGN, NuMA, p150^Glued^ (Fig. S3, *B* and *C*) and spindle orientation (Fig. S4). The peptide encoded by exons 20 to 21 plays a critical role in linking 4.1R with the LGN-NuMA-dynein/dynactin complex. Exons 20 to 21 of 4.1R are conserved among 4.1 family proteins. Whether other 4.1 members also participate in spindle orientation requires further investigation. The bifunctional properties of 4.1R indicate a delicate interplay between its role in astral microtubule stabilization and cortical attachment of the LGN-NuMA-dynein/dynactin complex. Both are required for proper mitotic spindle positioning.

Two Numb isoforms, Numb2 (−ex3/+ex9) and Numb4 (−ex3/−ex9), are expressed in erythroid cells. A switch from exon 9-containing to exon 9-excluding forms has been shown to be critical in neuronal lineage (30, 32) and endocrine lineage (31) development. Numb with exon 9 is predominantly expressed in progenitors, whereas forms without exon 9 are highly expressed in differentiated cells (7). Consistent with these reports, a switch of Numb2 in proliferating cells to Numb4 in differentiating cells occurred during erythropoiesis. Thus, exon 9 seems to play a role in proliferation and differentiation determination during erythropoiesis. It has also been shown that exon 3-containing forms promote proliferation, whereas exon 3-excluding forms promote differentiation (57). Since exon 3 is excluded from erythroid cells, the function of exon 3 may not strictly contribute to proliferation and differentiation in this lineage. T cell development and T cell activation occur normally in the absence of Numb, perhaps due to the expression of the related protein, Numb-like (58). Whether Numb-like protein is involved in erythropoiesis requires further analyses.

Several splicing factors have been implicated in exon 9 splicing regulation: both ASF/SF2 (6) and RbFox2 (59) negatively regulate exon 9 inclusion. We have shown that the expression of both factors is upregulated during erythroid differentiation (36, 60), suggesting that increased ASF/SF2 and RbFox2 may at least partly regulate exon 9 exclusion during erythroid differentiation. Thus, regulated exon 9 alternative splicing plays a critical role in mediating the temporal and/or spatial splicing decision of Numb during development of erythroid cells. It would be interesting to know how exon 3 exclusion is regulated and how the coordinated splicing regulation between exon 3 and exon 9 is mediated during differentiation.

We found an inverse expression relationship between increased Numb4 and decreased cleaved Notch1/Hes1 in differentiated MELCs (Fig. 7*B*). It has been reported that exon 9-excluding Numb suppresses Notch signaling and decreases Notch target gene expression, whereas the exon 9-containing form antagonizes the activity of the exon 9-excluding form and results in increased Notch target gene expression (61). Thus, changes in Numb isoform expression can modify the function of Numb as an inhibitor of the Notch signaling pathway during erythroid differentiation. We also showed that depletion of Numb increases cleaved Notch1 and Hes1 expression and correlates with reduced erythroblast population in induced MELCs (Fig. 7, *D* and *E*). These results suggest that erythroid differentiation is at least partly controlled by the interplay between Numb forms and the Notch signaling pathway. However, depletion of 4.1R, LGN, or NuMA also increased cleaved Notch1 and Hes1 (Fig. 7*D*) as well as reduced the erythroblast population (Fig. 7*E*) although each individual protein depletion did not affect the expression level of Numb (Fig. 4*B*). These results imply a more complicated relationship between 4.1R, LGN, or NuMA and Notch signaling that requires further investigation.

The most common distribution of Numb in asymmetric cell division is a crescent-like structure with cortical/membrane localization in many cell types. Different patterns of asymmetric Numb distribution and localization have been reported in erythroid cells. Numb in mouse GFP^+^KLSC cells localizes in the cytoplasm or with a crescent-like pattern in one daughter cell (10). Numb is colocalized with one centrosome in asymmetric cell division of differentiating CD34^+^ cells (37). In differentiating MELC, a round dot-shape of Numb is localized at the cell cortex with the 4.1R-LGN-NuMA complex in proximity to one of the spindle poles. Whether the shape and localization of Numb during erythroid asymmetric cell division are cell-type specific needs further investigation.

Members of the FERM family protein have been implicated in tumor progression (62). 4.1R was found to be involved in meningioma pathogenesis (63) and ependymal tumors (64). Despite extensive research on 4.1R’s role as a tumor suppressor, the mechanism by which 4.1R suppresses tumorigenesis has remained elusive. In this study, 4.1R deficiency leads to reduced asymmetric cell division and may instigate tumor growth by reducing differentiation and/or increasing proliferation. It is therefore tempting to speculate that spindle misorientation due to the disruption of 4.1R may contribute to tumor development and progression. Our study reveals that 4.1R is involved in a critical event by facilitating the stabilization of astral microtubules and proper localization of force generators, thereby providing a new perspective into the inner workings of spindle orientation.

## Experimental procedures

### Plasmid constructs

All DNA constructs were made using standard cloning procedures and confirmed by sequencing. Numb cDNAs were amplified from MELC RNA using oligo(dT) for RT and PCR with the sense primer mNumb-S (5′-ATGAACAAACTACGGCAAAGCTTCA-3′) and antisense primer mNumb-As (5′-CTAAAGTTCTATTTCAAATGTTTTCTG-3′) (GenBank accession number NM_001136075.2) for PCR. The products were cloned into TOPO-TA vector (Invitrogen). The identified representative species of mNumb isoforms were subsequently cloned in-frame with Flag in pcDNA3.1(+)-3Flag to form Numb1 (+ex3/+ex9), Numb2 (−ex3/+ex9), Numb3 (+ex3/−ex9), and Numb4 (−ex3/−ex9).

Human 4.1R rescue plasmids h4.1R^WT^ or h4.1R^−ex(20+21)^ lacking exons 20 and 21 (GenBank accession number NM_001166005.2) were cloned in frame with mCherry in pcDNA3.1(+)-mCherry to form h4.1R^WT^-mCherry and h4.1R^−ex(20+21)^-mCherry. h4.1R^80^ initiated from the second transcription start site was cloned in frame with Flag tag into pcDNA3.1(+)-Flag to form h4.1R^80^-Flag. Mouse 4.1R m4.1R^WT^-mCherry or m4.1R^−ex(20+21)^-mCherry BAC clones were constructed as follows. DH10 *E. coli* harboring mouse 4.1R BAC clone RP24-99H4 was obtained from BACPAC Resources. Cloning steps were done according to the Counter-Selection BAC Modification kit (version 3.1; Gene Bridges). The C-terminal GFP-LAP tag vector was a gift from the Hyman Laboratory (Molecular Cell Biology and Genetics, Max Planck Institute). Recombineering replaced the m4.1R stop codon or the last codon at the 3′ end of exon 19 (GenBank accession number NM_183428.3) with the GFP-LAP tag yielding the m4.1R^WT^-GFP-LAP or m4.1R^−ex(20+21)^-GFP-LAP construct, respectively (65). The GFP region was exchanged with mCherry to form m4.1R^WT^-mCherry or m4.1R^−ex(20+21)^-mCherry. BAC DNA was purified by following NucleoBond BAC 100 maxi prep protocol (Clontech). Preparation of cells for electroporation was performed at 4 °C and electroporation voltage was kept consistent at 1.8 kV using a MicroPulser Electroporator (Bio-Rad Laboratories).

### Cell culture and transfection

Human cord blood CD34^+^ cells were purchased from Stemcell Technologies and terminal erythroid differentiation was performed as described (33). MELC and HeLa cells were cultured in Dulbecco modified Eagle medium (DMEM, Corning) supplemented with 10% fetal bovine serum (FBS, Sigma) and Penicillin-Streptomycin (Gibco).

For gene depletion in MELCs, 2.5 ml of 1 × 10^5^ cells/ml were transfected with complex containing 5 μl RNAiMAX (Invitrogen) and 150 ρmol siRNA according to the manufacture’s protocol. Cells were induced with 2% DMSO in DMEM medium. To express h4.1R^WT^-mCherry or h4.1R^−ex(20+21)^-mCherry, MELCs were transfected with Lipofectamine LTX (Invitrogen) according to the manufacturer’s protocol. To inhibit Notch signaling, DAPT (50 μM, Calbiochem) was added to the medium. To depolymerize the microtubules, cells were treated with 100 ng/ml nocodazole (Sigma-Aldrich). To depolymerize the actin filament, cells were treated with Latrunculin A (0.1 μg/ml) (Thermo Fisher Scientific).

HeLa was treated with transfection complexes containing 3 μl Oligofectamine (Invitrogen) and 60 ρmol siRNA for gene depletion according to the manufacture’s protocol. Histone H2B-GFP HeLa cell line was created by transfection with H2B-GFP (Addgene) and selection of stable cell lines was performed with the addition of 800 μg/ml of G-418. BAC constructs were transfected into HeLa cells in 6-cm dishes with Effectene (Qiagen) according to the manufacturer's protocol. To isolate clonal cell lines expressing m4.1R^WT^-mCherry or m4.1R^−ex(20+21)^-mCherry, cells were plated at low density and isolated colonies were grown in the presence of G418. The level of transgene expression was determined by western blotting.

### siRNA duplexes

siRNA duplexes were purchased from Dharmacon. A siRNA duplex targeting GFP was used as control (Ctrl siRNA 5′-GGCUACGUCCAGGAGCGCACC-3′) (66). Previously reported siRNA duplexes were used to deplete human 4.1R (h4.1R 5′-CCAGCACAGUUAACAGAAGACAUAA-3′) (28), mouse 4.1R (m4.1R 5′- GAAGGUCUGUGUGGAGCAU-3′), human LGN (hLGN 5′-GCUGCAGUUCAAGUUGGAACU-3′) (67), mouse LGN (mLGN 5′- GGUCUAAGCUACAGCACAAAU-3′) (68), human NuMA (hNuMA 5′-GGCGUGGCAGGAGAAGUUC-3′) (69), mouse NuMA (mNuMA 5′-GCCAGAUGGAUCGAAAGAUU-3′) (70), human and mouse dynein-heavy chain (dynein 5′-AGGCUUUAACCAAGCAGAUAA-3′) (71), mouse Numb (mNumb 5′-GCACCUGCCCAGUGGAUCC-3′) (72), human p150^Glued^ (hp150^Glued^ 5′-GCCUUGAACAGUUCCAUCAUU-3′) (73), and human Gαi3 (hGαi3 5′-CCGAAUGCAUGAAAGCAUG-3′) (40).

### RT-PCR analyses

RT-PCR analysis of expression of alternatively spliced exons was performed using a limiting cycle amplification protocol that obtains the PCR product within its linear range (34). RNAs from MELC or CD34^+^ cells were reverse transcribed using the oligo(dT) primer. PCRs for mouse Numb were performed with mEx2-S (5′-TACCTCGGCCACGTAGAAGT-3′) and mEx4-As (5′-TCTCGTCCACAACTCTGAGC-3′) for exon 3 and with mEx8-S (5′-CTCTGAGGACCCCTTCTCCT-3′) and mEx10-As (5′-GGTCAGCTTCAGAGGGAGTG-3′) for exon 9. PCRs for human Numb (NM_001005743.1) were performed with hEx5-S (5′-TACCTTGGCCATGTAGAAGT-3′) and hEx7-As (5′-TTTCATCCACAACTCTGAGT-3′) for exon 6 and with hEx11-S (5′-ACCTGAGGACCCCTTCTCAT-3′), and hEx13-As (5′-GGTCGGCCTCAGAGGGAGTA-3′) for exon 12. PCR products were fractionated on 2% agarose gels or 5% DNA polyacrylamide gels and quantified using analytic software from a ChemiImagerTM 5500 System (Alpha Innotech Co). Ex9 inclusions were calculated as the percent of exon 9 inclusion (Ex9)/total products (set as 100%).

### Antibodies

The antibodies against the following proteins were used as follows: EPB41 (rabbit, Sigma; 22, 23), Giα3 (rabbit, Millipore), NuMA (rabbit, Cell Signaling Technology; rabbit, Abcam), LGN (rabbit, EMD Millipore Corp; Dr Quansheng Du, Georgia Regents University), p150^Glued^ (mouse, BD Transduction Laboratories), dynein (IC 74.1, Chemicon), Numb (mouse, Millipore; rabbit, Abcam; Rabbit, Novus Biologicals), Nestin (mouse, Developmental Studies Hybridoma Bank), α-Hemoglobin (rabbit, Santa Cruz biotechnology), Notch1 (mouse, BioLegend), Hes1 (mouse, OriGene Technologies), pericentrin (rabbit, Covance), β-actin (rabbit, GeneTex; AC-74, Sigma-Aldrich), γ-tubulin (T5192, rabbit, Millipore Sigma; clone GTU88, Sigma-Aldrich), α-tubulin (mouse, Calbiochem; mouse, Sigma; clone YL1/2; EMD Millipore), GAPDH (mouse, Sigma), mCherry (Rabbit, BioVision), Flag (mouse, Sigma), Ter-119 APC (Rat, eBioscience), CD71 FITC (Rat, BD Pharmingen).

### Coimmunoprecipitation and immunoblotting

The MELC or HeLa cells were extracted in cell lysis buffer (50 mM HEPES, pH7.5, 150 mM NaCl, 1% NP-40) and protease inhibitor cocktail (Roche) for 30 min and centrifuged at 15,100*g* for 10 min. The supernatant was precleared with protein A-Sepharose (Roche) beads for 30 min at 4 °C. The antibodies were bound to the beads containing 0.1% BSA, 150 mM NaCl, 20 mM Tris-HCl pH 8.0, and 0.05% Tween 20 by rocking at 4 °C overnight. Cell lysates or immunoprecipitates were analyzed by 10% SDS-PAGE and electrotransferred onto a polyvinylidene difluoride (PVDF) (Millipore) or nitrocellulose membrane (Maine Manufacturing, LLC). The detection of 4.1R and its associated proteins was carried out by immunoblotting with its respective antibody diluted in either 4% milk in TBST (20 mM Tris-HCl, pH 7.6, 140 mM NaCl, 0.1% Tween-20) or in antibody enhancer diluent (Amresco, Inc), and developed using an ECL detection kit (Amersham Pharmacia). The presence of exogenously expressed mCherry-tagged proteins was detected with anti-mCherry Ab. VeriBlot secondary antibodies (ab131366 or ab131368, Abcam) were used for coimmunoprecipitation western blot analyses.

### Indirect immunofluorescence and imaging

For immunolabeling experiments, HeLa cells were grown on poly-d-lysine-coated coverslips and MELC cells were grown on fibronectin coated coverslips. MELC suspension was pelleted by centrifugation, washed with PBS, and pelleted again. Cells on coverslip or suspension were fixed in PBS containing 4% paraformaldehyde for 15 min and permeabilized in 0.5% Triton X-100 in PBS for 10 min at room temperature. MELC suspension cells were then washed in PBS, resuspended in 10% BSA in PBS, cytospun on coverslips, and dried for 10 min at room temperature. Cells on coverslips were blocked in 10% goat serum for 30 min and exposed to various primary antibodies for 1 h at 37 °C, followed by Alexa Fluor 568 goat anti-mouse, Alexa Fluor 568 goat anti-rabbit, Alexa Fluor 488 goat anti-mouse, Alexa Fluor 488 goat anti-rabbit secondary antibody, or Alexa Fluor 488 Phalloidin (Invitrogen) at 37 °C for 1 h. All samples were counterstained with 4,6-diamidino-2-phenylindole (DAPI) (Sigma-Aldrich).

Images were collected with a Yokogawa CSU-22 spinning disk confocal mounted on a Zeiss Axiovert microscope using 408, 488, and 561 nm laser light. A series of 0.25 μm optical sections was acquired using a ×100 1.4 NA Plan Apo objective with an Orca ER CCD camera (Hamamatsu Photonics). *Z*-stacks were collected with a 0.25 μm step size with pinhole at 1 Airy unit. All image processing was performed by using SlideBook and Metamorph software. The distance between signals and fluorescence intensities of the signals were analyzed with the ImageJ (http://rsb.info.nih.gov/ij/download.html). The 3D angle tool of Image J was used to measure the angles in 3D. Some samples were viewed with a Zeiss Axiovert 200M inverted microscope or a Zeiss Axio Imager.Z1 microscope (Zeiss, Inc). The images were collected using SlideBookTM 4.0 or AxioVision Rel. 4.8 software and processed using Photoshop software (Adobe Systems, Inc).

For live cell imaging, the medium was replaced with a CO_2_-independent medium supplemented with 10% fetal bovine serum, penicillin-streptomycin, and L-glutamine (Invitrogen) and covered with mineral oil immediately before analysis. Cells were maintained at 37 °C using a heated stage. Images of cells expressing H2B-GFP were collected under an inverted microscope (Olympus IX81) using a 40 × NA 1.4 PlanApo objective. The acquisition parameters, including exposure, focus, and illumination were controlled by the Elements software (Universal Imaging). Single focal plane images were collected by a camera at 3-min intervals. The *z*-stacks were projected using the NIS-elements software. All subsequent analyses and processing of images were performed using the NIS-elements software (Nikon Instruments Inc).

### Quantification of cortical intensity and astral microtubule intensity

Cortical intensity of LGN, NuMA, or p150^Glued^ was quantified from fluorescence micrographs carried out with ImageJ software. In brief, the area outline of the cell was drawn as the area of the cell periphery and the regions of cortical bipolar crescent-shaped area labeled by LGN, NuMA, or p150^Glued^ was drawn as labeled area. Three cells/field from control or each siRNA knockdown (ten fields for each sample; total 30 cells) were measured for both cell periphery area and each protein labeled area. The percentage of cortical intensity was obtained by the ratio of each protein labeled area over the total area of the cell periphery.

To analyze the relative astral microtubule intensity, the fluorescence intensities of total and spindle microtubules were measured from fluorescence micrographs using the ImageJ software. The ratio of fluorescence intensity (total intensity − spindle intensity)/spindle intensity for the control and each protein knockdown sample was calculated from 30 cells.

### Spindle orientation analysis

Spindle orientation analysis was described previously (43). The linear distance (X μm) and the vertical distance (Y μm) between the two poles of the mitotic spindles were measured by taking Z-stack images from 0.25 μm-thick sections of a mitotic cell stained with an anti-γ-tubulin Ab and DAPI. The angle between the axis of a mitotic spindle and that of the substrate surface was calculated with trigonometric function.

### Spindle orientation assay with CYTOO chips

HeLa or HeLa cells stably expressing m4.1R^WT^-mCherry or m4.1R^−ex(20+21)^-mCherry were treated with h4.1R siRNA. Approximately 24 h after siRNA treatment, cells were plated on L-shaped, fibronectin-micropatterned chips (CYTOO). Approximately 60,000 cells were placed on a CYTOO chip in a 35 mm culture dish. After 1 h, floating cells not attached to the micropatterns were removed. Cells were fixed with 4% paraformaldehyde and permeabilized with 0.5% Triton X-100 in PBS and stained for γ–tubulin.

### Cell pair assays

To examine how Numb protein is distributed when cells divide, we developed the “cell pair assay.” MELCs were induced with DMSO and plated at low density (300–500 cells/well) that resulted in single cell in Cultureslides (BD) coated with ClonaCell FLEX (Stemcell Technologies). Most of the cells completed the first round of divisions within 24 h. The cells were immunostained and examined for the distribution of Numb, Nestin, and α-Hemoglobin with its respective Ab.

### Flow cytometry analysis

Control or siRNA-treated MELC was induced to erythroid differentiation with the addition of 2% DMSO to the medium. After washing with PBS containing 2% FBS, cells were immunostained with FITC-CD71 (0.5 μg/10^6^ cells) and APC-TER119 (0.5 μg/10^6^ cells) Abs. The 7-aminoactinomycin D (7-AAD, Invitrogen) was added to exclude dead cells from the analysis. Cells were fixed in methanol and stained with propidium iodide and analyzed for cell cycle profile. For the apoptosis analysis, cells were stained for PI and FITC conjugated Annexin V according to the manufactures protocol (FITC Annexin V Apoptosis Detection Kit, BD) and analyzed by flow cytometry.

### Statistical analysis

Two or three samples were performed in each experiment. Each experiment was repeated three times. Data were analyzed using GraphPad Prism 8 software (GraphPad Software, Inc). All values were expressed as mean ± SD. Statistical significance of quantitative data was determined by Student’s *t* test. The level of significance difference was determined at *p*∗∗∗ < 0.0005; *p*∗∗ < 0.005.

## Data availability

All data are contained within the manuscript.

## Supporting information

The article contains supporting information.

## Conflict of interest

E. J. B., Jr serves on the Boards of Directors of Deci0hera Pharmaceuticals, Candel Therapeutics, Renovacor, and F-Star Therapeutics, LLC, and advisory boards of Kernel Therapeutics, Autoimmune Solutions, Inc, and Riverside Partners, and is Executive Director of the Cure Sickle Cell Initiative sponsored by 10.13039/100000002NIH. He receives stipends and/or stock options for these activities, none of which are relevant to the content of this manuscript.
