# Supplementary material for: Multifunctional protein 4.1R regulates the asymmetric segregation of Numb during terminal erythroid maturation
Source: J Biol Chem. 2021 Aug 6;297(3):101051. doi: 10.1016/j.jbc.2021.101051 (PMC8408529; doi:10.1016/j.jbc.2021.101051)
Supplement: Supplemental Figures S1–S4 [file mmc1.pdf]

Protein 4.1R regulates asymmetric segregation of Numb during terminal erythroid maturation

Shu-Ching Huang, Long V. Vu, Faye H. Yu, Dan T. Nguyen, Edward J. Benz Jr.

Supplemental Data

Figures S1 to S4

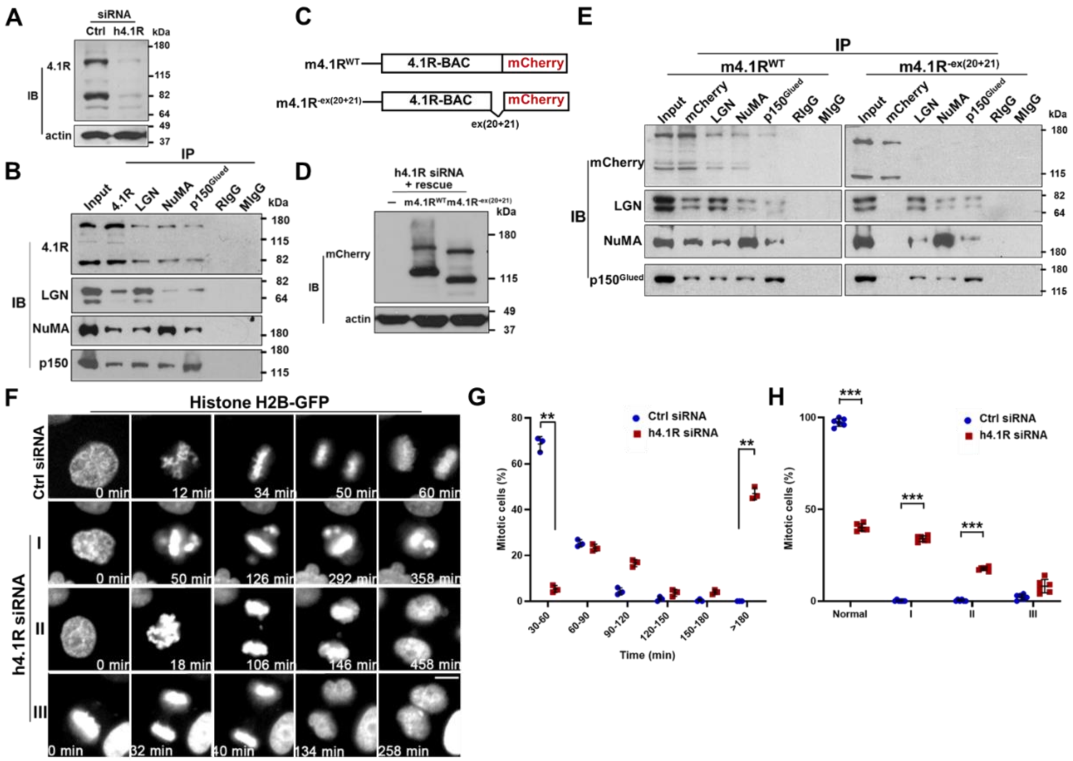

Figure S1. 4.1R associates with the LGN-NuMA-p150^{Glued} complex through its interaction with NuMA and 4.1R depletion induces a mitotic block at prometaphase/metaphase of HeLa cells. HeLa cells depleted of 4.1R were transfected with rescue construct m4.1R^{WT}-mCherry or m4.1R$^{-ex(20+21)}$-mCherry and analyzed for the ability of 4.1R forms to associate with LGN-NuMA-p150^{Glued} complex. **A,** Immunoblots for 4.1R expression in control or h4.1R siRNA knockdown cells with an anti-4.1R Ab. Actin served as a loading control. Molecular mass markers (kDa) are provided. **B,** Association of the endogenous 4.1R, LGN, NuMA, and p150^{Glued} proteins in co-immunoprecipitation assays. **C,** Rescue constructs m4.1R^{WT}-mCherry and m4.1R$^{-ex(20+21)}$-mCherry with native 4.1R promoter. **D**, Efficiency of 4.1R depletion and rescue construct expression. Cell extracts of h4.1R siRNA treated and transfected with or without m4.1R^{WT}-mCherry or m4.1R$^{-ex(20+21)}$-mCherry were blotted with an anti-mCherry Ab. Actin served as a loading control. Molecular mass markers (kDa) are provided. **E**, Association of 4.1R, LGN, NuMA, and p150^{Glued} proteins in co-immunoprecipitation assays. m4.1R^{WT}-mCherry or m4.1R$^{-ex(20+21)}$-mCherry cell lysates were precipitated (IP) with an anti-mCherry, anti-LGN, anti-NuMA, anti-p150^{Glued}, RIgG, or MIgG Ab. The input extracts and immunoprecipitates were examined by immunoblotting (IB) with its corresponding Ab. Molecular mass markers (kDa) are provided. **F,** Prolongation of cell cycle and aberrations of chromosomes due to 4.1R depletion. HeLa cells stably expressing Histone H2B-GFP and transfected with a control or h4.1R siRNA were imaged by time-lapse fluorescence microscopy. Three types of aberrant chromosome behaviors were observed. I: cells that underwent apoptosis; II: anaphase was initiated in the presence of non-aligned chromosomes; III: anaphase was initiated but chromosomes were not able to separate successfully during cytokinesis. Bars, 5 µm. **G,** Quantification of durations of mitotic phase in cells treated with control or h4.1R siRNA as indicated. n = 50 cells per group with two replicas in each experiment. Three independent experiments were analyzed. **H,** Quantification of chromosome dynamics in control or h4.1R depleted cells. I-III correspond to chromosome aberrations observed in **F**. n = 50 cells per group with two replicas in each

experiment. Three independent experiments were analyzed. All values are expressed as means ± SD. Student *t* test for all graphs. ***$p < 0.0005$; **$p < 0.005$.

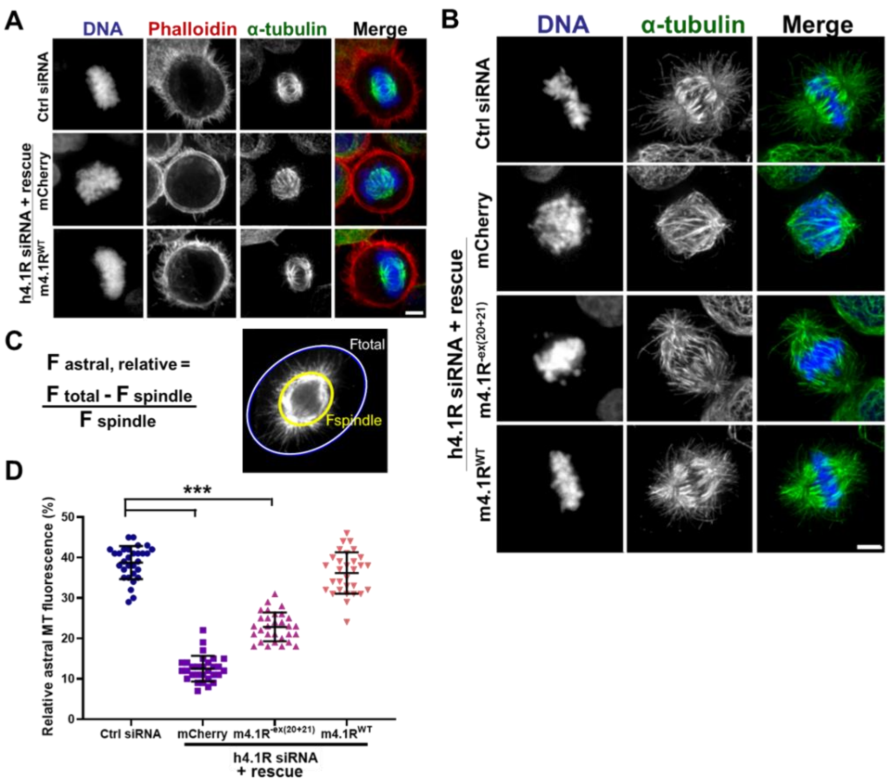

Figure S2. Interaction between 4.1R and NuMA promotes retraction fiber formation and the stability of astral microtubules. HeLa cells depleted of 4.1R were transfected with rescue construct m4.1R^{WT}-mCherry or m4.1R$^{-ex(20+21)}$-mCherry and analyzed for the effect of 4.1R on retraction fiber formation and astral microtubule stability. **A**, 4.1R is required for retraction fibers formation. Immunofluorescent staining of HeLa cells transfected with control or h4.1R siRNA co-expressing mCherry or m4.1R^{WT}-mCherry followed by staining with anti–α-tubulin Ab (green), phalloidin (red), and DAPI (blue). Metaphase cells were imaged. Scale bars, 5 µm. **B**, 4.1R stabilizes astral microtubules. Immunofluorescent staining of HeLa cells transfected with control or h4.1R siRNA in the presence of mCherry, m4.1R$^{-ex(20+21)}$-mCherry, or m4.1R^{WT}-mCherry followed by staining with an anti–α-tubulin Ab (green) and DAPI (blue). Metaphase cells were imaged. Scale bars, 5 µm. **C**, Schematic depicting the formula for measuring relative astral microtubule fluorescence. Total and spindle microtubule intensities were quantified from fluorescence micrographs using ImageJ. **D**, Quantification of relative astral microtubule fluorescence in cells treated as in **B**. n = 30 cells per group in each experiment. Three independent experiments were analyzed with similar results. All values are expressed as means ± SD. Student *t* test for all graphs. ***$p < 0.0005$.

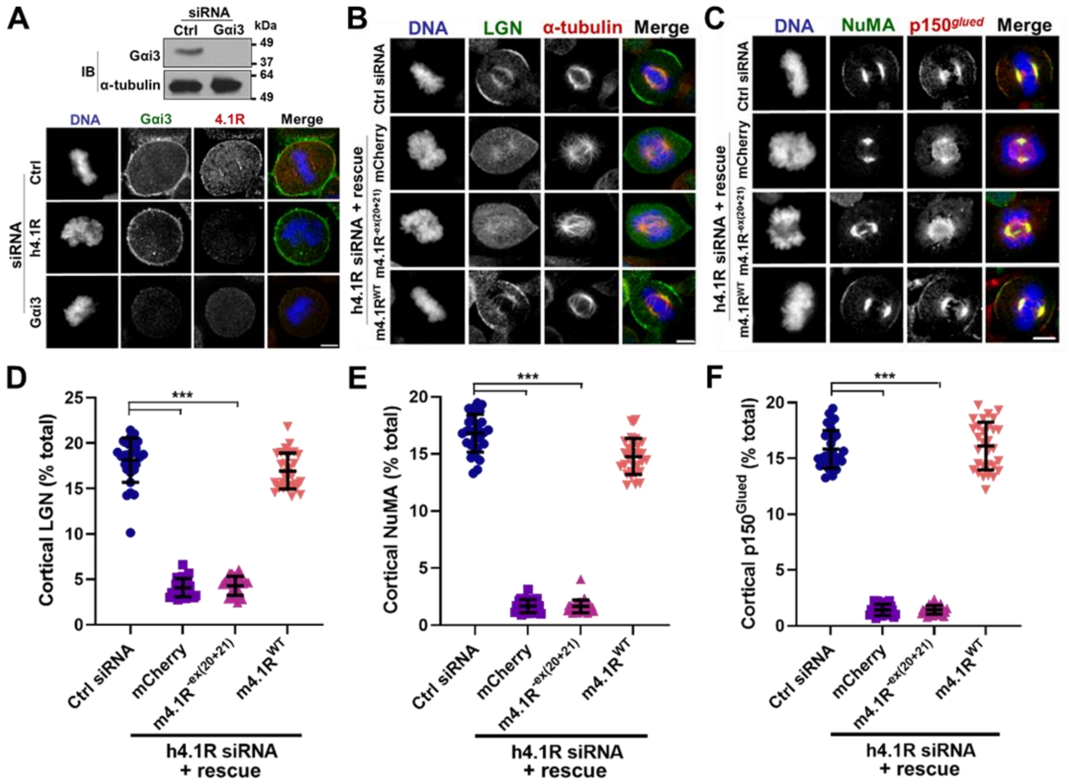

Figure S3. Loss of 4.1R impairs the cortical localization of LGN, NuMA, and p150^{Glued}. A, Gαi3 depletion impairs 4.1R cortical localization. Upper panel, Efficiency of Gαi3 depletion. Cell extracts of control and Gαi3 siRNA treated were blotted with an anti-Gαi3 Ab. α-tubulin served as a loading control. Molecular mass markers (kDa) are provided. Lower panel, Immunofluorescent staining of HeLa cells transfected with control siRNA, h4.1R siRNA, or Gαi3 siRNA, followed by staining with anti-Gαi3 (green) and anti-4.1R (red) Abs and DAPI (blue). Scale bar, 5 μm. **B-C,** 4.1R is required for cortical localization of LGN, NuMA, and p150^{Glued}. Immunofluorescent staining of cells transfected with control or 4.1R siRNA co-expressing mCherry, m4.1R$^{-ex(20+21)}$-mCherry, or m4.1R^{WT}-mCherry followed by staining with anti-LGN (green) and anti–α-tubulin Ab (red) (**B**) or with anti-NuMA (green) and anti-p150^{Glued} Ab (red) (**C**). DAPI (blue). Scale bar, 5 μm. **D-F,** Quantification of cortical localization of LGN (**D**), NuMA (**E**), and p150^{Glued} (**F**) from panels **B** and **C**. Total cortical and LGN, NuMA, or p150G^{lued} cortical intensities were quantified from fluorescence micrographs using ImageJ. Cortical intensity of each protein expressed as % of total cortical intensity was calculated as described in "Experimental Procedures." n = 30 cells per group in each experiment. Three independent experiments were analyzed with similar results. All values are expressed as means ± SD. Student *t* test for all graphs. ***$p < 0.0005$.

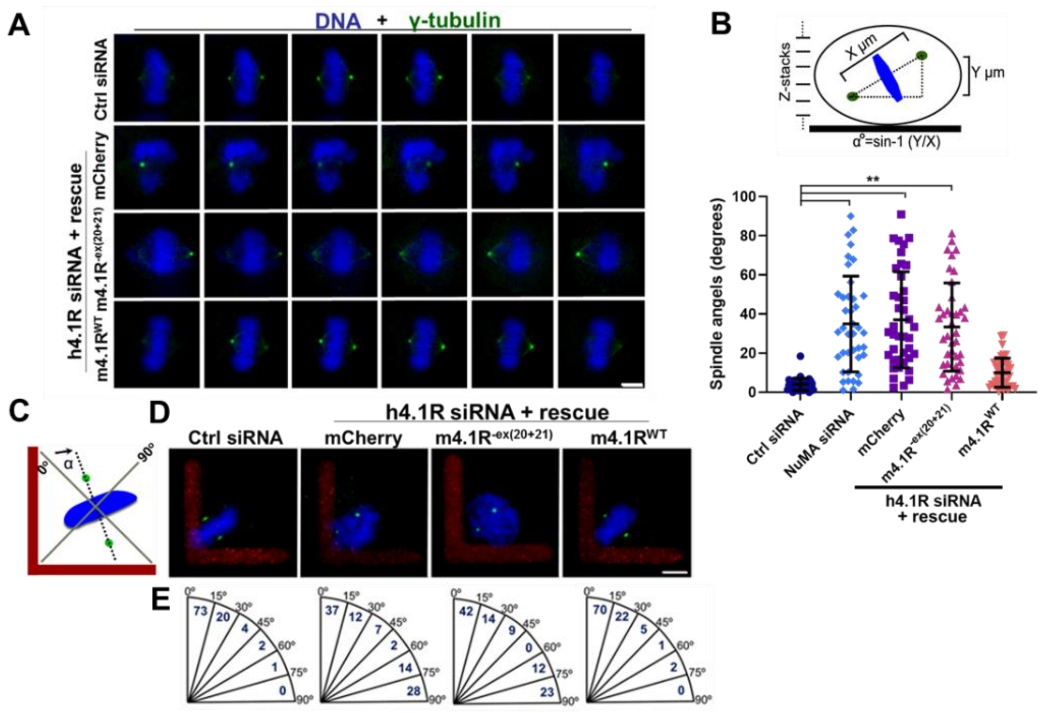

Figure S4. Loss of 4.1R leads to spindle mis-orientation. A, Representative Z-stack images of Hela cells transfected with control or 4.1R siRNA co-expressing mCherry, m4.1R$^{-ex(20+21)}$-mCherry, or m4.1R^{WT}-mCherry, followed by staining with an anti–γ-tubulin Ab (green) and DAPI (blue). Metaphase cells were imaged. Scale bar, 5 µm. **B,** Spindle orientation analysis. Upper panel, Scheme depicting spindle angle (α) measurement. Lower panel, Distribution of spindle angles in cells treated with a control siRNA, NuMA siRNA, or h4.1R siRNA co-expressing mCherry, m4.1R$^{-ex(20+21)}$-mCherry, or m4.1R^{WT}-mCherry. n = 40 cells per group in each experiment. Three independent experiments were analyzed with similar results. All values are expressed as means ± SD. Student *t* test for all graphs. $^{**}p < 0.005$. **C,** Schematic representation of mitotic spindle geometry of the L-shaped micropattern. The centrosome is shown in green and chromosomes in blue. Spindle position is determined as an angle, as depicted, with 0° being defined as parallel to the hypotenuse of the L. **D,** Representative mitotic cells on an L-shaped fibronectin micropattern (red) stained with an anti-γ-tubulin (green) Ab. Note that the spindle axis along the L-shaped micropatterned fibronectin substrate orients along the hypotenuse of the L-shape during mitosis. Bar, 5 µm. **E,** Frequency of angular (0 – 90°) distributions of spindle orientation as in **D** for cells treated with control or h4.1R siRNA co-expressing mCherry, m4.1R-$^{ex(20+21)}$-mCherry, or m4.1R^{WT}-mCherry. n = 100 cells per group. Three independent experiments were analyzed with similar results.
